# Multifunctional Luminescent Solar Concentrator Integrating Optical Thermometry Based on PMMA/Eu^III^-Complex Films for Smart Windows

**DOI:** 10.1021/acsami.6c03810

**Published:** 2026-07-06

**Authors:** Felipe Canisares, Mariana V. Corrêa, Juliana Izidoro, Paulo R. S. Santos, João H. A. Neto, Javier A. Ellena, Airton G. Bispo-Jr, Maria Claudia F. C. Felinto, Oscar L. Malta, Hermi F. Brito

**Affiliations:** † Department of Fundamental Chemistry, Institute of Chemistry, 28133 University of São Paulo (USP), São Paulo, São Paulo 05508-000, Brazil; ‡ Scientific Learning Program, 119500 Dante Alighieri School, São Paulo, São Paulo 01420-002, Brazil; § São Carlos Institute of Physics, University of São Paulo (USP), SãoCarlos, São Paulo 13566-590, Brazil; ∥ Nuclear and Energy Research Institute, São Paulo, São Paulo 05508-900, Brazil; ⊥ Federal University of Pernambuco, Recife, Pernambuco 50670-901, Brazil

**Keywords:** multifunctional material, solar concentrator, sunlight excitation, optical conversion efficiency, thermometry

## Abstract

Multifunctional luminescent materials capable of simultaneously harvesting solar energy and monitoring environmental conditions are promising for next-generation smart windows. Here, we report transparent poly­(methyl methacrylate) (PMMA) films doped with the Eu^III^-based (Et_4_N)­[Eu­(nta)_4_] complex (nta^–^: 4,4,4-trifluoro-1-(naphthalen-2-yl)­butane-1,3-dionate; Et_4_N^+^: tetraethylammonium), designed to function as both luminescent solar concentrators (LSCs) and luminescent temperature probes. The nta^–^ ligand enables efficient sunlight absorption in the near-ultraviolet spectral range and sensitization of the Eu^III^ luminescence, affording overall emission quantum $\left(Q_{Eu}^{L}\right)$ yields of up to 50% and sensitization efficiencies above 90%. The resulting PMMA films remain highly transparent (90–100% transmittance) and display homogeneous dopant dispersion, yielding intense red Eu^III^ emission upon exposure to sunlight and effective waveguiding. When coupled to a silicon photovoltaic cell and exposed to daylight, the PMMA:2%Eu^III^ film enhances current generation relative to an uncoated glass substrate, confirming its LSC capability. Moreover, the Eu^III 5^D_0_ lifetime is gradually quenched from 280 to 420 K (maximum relative thermal sensitivity of 4.7% K^–1^ at 397 K), indicating that the film can operate as a temperature probe in locations exhibiting elevated temperatures (e.g., tropical countries, rooftops, parking lots). These results establish Eu^III^-doped PMMA films as promising dual-function platforms for transparent, energy-harvesting smart windows with integrated optical temperature monitoring.

## Graphical abstract

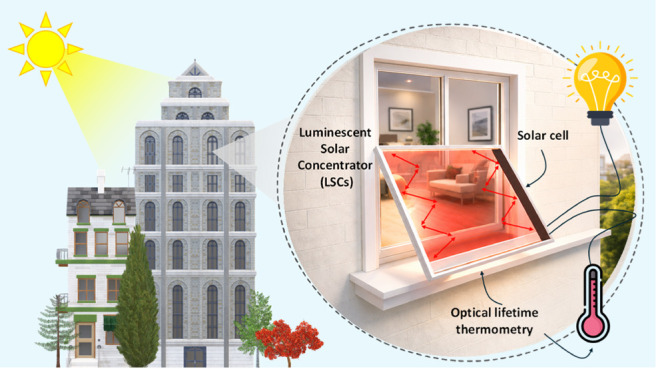

## Introduction

The growing demand for energy-efficient building technologies has stimulated the development of multifunctional optical materials capable of lowering energy consumption while providing additional functional benefits to architectural elements.^1^ Enhancing the energy performance of buildings is especially critical in densely populated and industrialized regions, where urban infrastructure accounts for nearly 40% of global carbon emissions.^2,3^ Smart windows capable of harvesting sunlight to generate electricity have emerged as an attractive strategy for enhancing thermal regulation,^4^ daylight control,^5^ and overall sustainability in urban infrastructure.^6^ Yet, although systems based on electrochromic,^7^ thermochromic,^8^ or photochromic^9^ mechanisms have demonstrated practical utility, most remain limited to passive light modulation and offer limited capacity for energy harvesting or integrated sensing. This limitation highlights the need for advanced materials that can simultaneously manage solar radiation and actively contribute to energy generation and environmental monitoring.

As an alternative, transparent^10^ or semitransparent^11^ platforms capable of converting sunlight into electricity have attracted significant attention for use as smart windows. Among these technologies, luminescent solar concentrators (LSCs) stand out due to their ability to absorb near-ultraviolet (UV) solar radiation that is not efficiently harvested by silicon photovoltaics, re-emit it at longer wavelengths that better match the maximum efficiency of silicon devices, and guide the emitted light toward photovoltaic cells located at the device edges.^12^ This architecture enables the use of large-area and visually transparent surfaces without compromising aesthetics or building illumination.^13^ Polymers such as poly­(methyl methacrylate) (PMMA) incorporated with luminescent activators are promising for LSCs due to their ease of processing, transparency, scalability, mechanical stability, and resistance to photodegradation.^14^ This level of spectral and aesthetic control surpasses that of conventional thin-film photovoltaic technologies, including silicon,^15^ cadmium telluride (CdTe),^16^ and copper indium gallium selenide (CIGS) systems.^17^

The performance of LSCs depends on several features of the luminescent activators, such as emission quantum yield, large Stokes shift, strong absorption within the near-UV-to-blue spectral window, emission spectra that match the maximum efficiency of silicon photovoltaics, and stability under solar irradiation. Lanthanide­(III)-based complexes, particularly those containing Eu^III^, meet most of these requirements since they present a large pseudo-Stokes shift and strong molar absorptivity within the near-UV-to blue spectral region associated with the well-spread antenna effect. The intraconfigurational 4*f–4f* electronic transitions also have long excited-level lifetimes (∼10^–3^ s) and narrow emission bands in the red spectral region, where silicon photovoltaics present large external quantum efficiency.^18^ Besides the photophysical features for LSCs, the excited-level lifetime of Eu^III^ is sensitive to environmental changes, enabling optical thermometry, which is a valuable multifunctionality for smart windows.^19^ In this context, buildings equipped with LSCs that support optical thermometry could respond more intelligently to environmental changes by integrating with the building’s thermal management system.

The integration of lanthanide­(III)-based LSCs with real-time temperature monitoring in a single polymeric platform remains largely unexplored. Existing studies generally focus either on optimizing luminescent efficiency for solar concentration^20^ or on exploiting thermometric capabilities^21,22^ but rarely investigate their combination in a transparent film. Alyami et al.^23^ reported coumarin–CdTe@PMMA nanohybrid films as dual-function LSC windows, enabling simultaneous light harvesting and autonomous temperature sensing. Correia et al.^24^ replaced environmentally problematic quantum dots with bacteriochlorophyll (BChl) dispersed in a styrene–ethylene–butylene–styrene (SEBS) matrix, yielding red/near-infrared (NIR)-emissive LSCs. Yet, critical aspects such as dopant dispersion, long-term photostability, and the balance between transparency and optical performance require further investigation to advance this technology.

To address this exciting challenge, our strategy is to design PMMA films doped with an europium­(III) complex projected to operate simultaneously as a luminescent solar concentrator and an optical temperature sensor (Figure 1). To achieve such a task, the tetrakis­(β-diketonate) (Et_4_N)­[Eu­(nta)_4_] complex (nta^–^: 4,4,4-trifluoro-1-(2-naphthyl)­butane-1,3-dionate, Et_4_N^+^: tetraethylammonium) was employed as luminescent activator in the LSC. The conjugated structure of the ligand framework is predicted to convert near-UV-to-blue sunlight into red light, matching the silicon photovoltaic maximum absorption. Additionally, thermal quenching of the Eu^III 5^D_0_ emitting level lifetime provides a reliable mechanism for real-time temperature assessment. By tailoring the structure of the Eu^III^ complex and optimizing its incorporation into PMMA, we carefully investigated how the photophysical features of the films can be tuned to improve light conversion and thermometry capability without compromising transparency. Thus, this dual-function platform represents a significant step toward the development of next-generation smart windows for sustainable energy management and intelligent building technologies.

**Figure 1:**
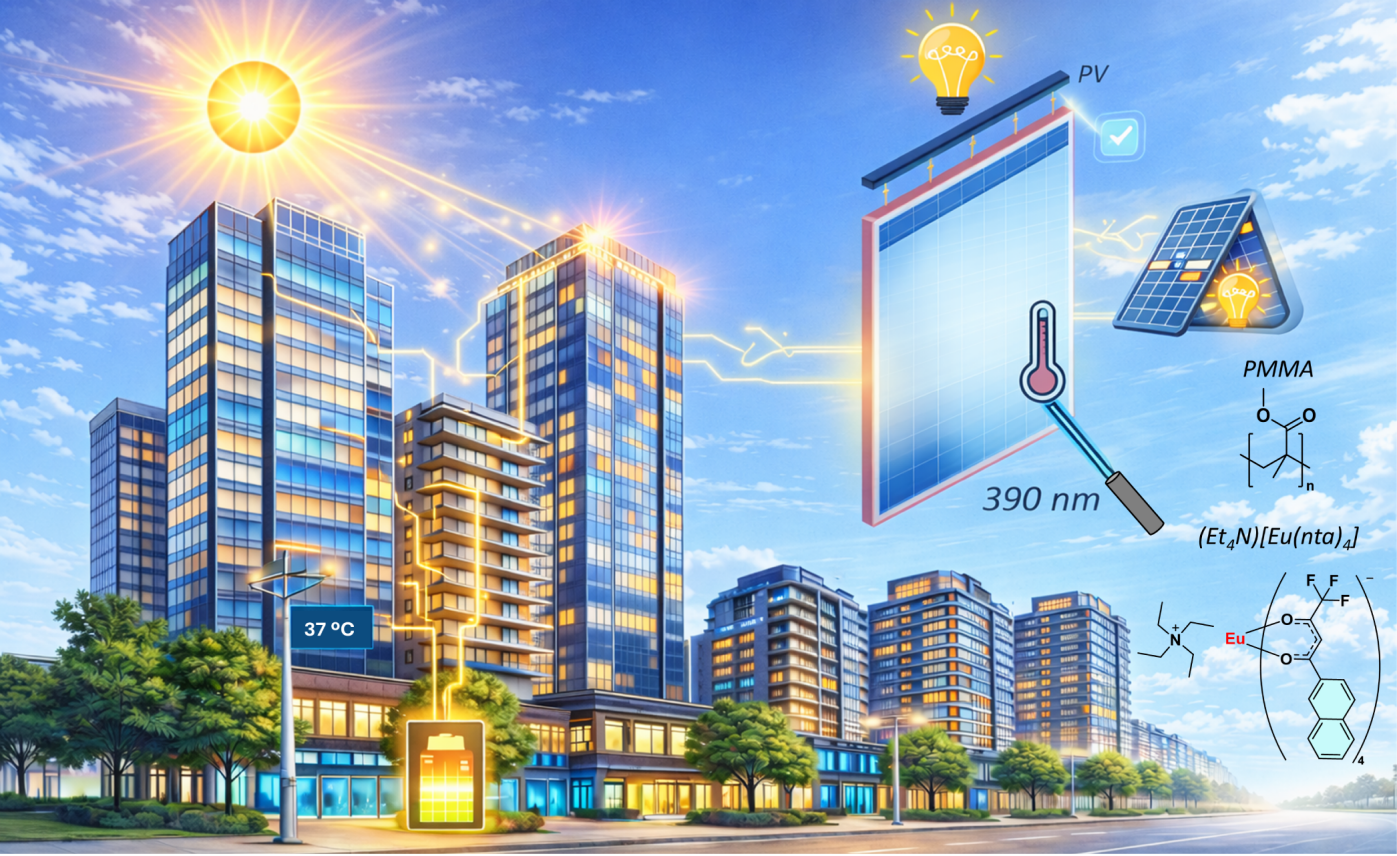
Smart buildings equipped with multifunctional windows coated by the PMMA film doped with the (Et_4_N)­[Eu­(nta)_4_] complex. This architecture enables the integration of solar energy harvesting, via luminescent solar concentrators, and luminescent temperature sensing within a single platform.

## Experimental Section

No uncommon hazards were noted in any of the procedures described below.

### Synthesis of the (Et_4_N)­[Ln­(nta)_4_] Complex (Ln: Eu^III^ or Gd^III^)

The (Et_4_N)­[Ln­(nta)_4_] complexes (Ln = Eu^III^ or Gd^III^) were synthesized as reported elsewhere.^25^ For that, LnCl_3_ was dissolved in a methanol/water mixture, and a methanolic solution of deprotonated nta^–^ (5:1 nta^–^:Ln^III^, using NaOH) was added dropwise. Subsequently, a methanolic solution of tetraethylammonium bromide (Et_4_NBr) was introduced. The mixture was stirred at 60 °C for 3 h. The resulting precipitate was filtered, dried in a desiccator, and recrystallized by layering hexanes over a dichloromethane solution to yield transparent crystals. Detailed characterization data are provided in Supplementary Note S1.

### (Et_4_N)­[Eu­(nta)_4_]

FTIR (cm^–1^), Figure S1: 1612 (vs), 1569 (w), 1526 (m), 1507 (m), 1456 (s), 1428 (m), 1383 (w), 1347 (w), 1288 (vs), 1247 (w), 1179 (s), 1132 (vs), 1071 (w), 1055 (w), 1051 (w), 1027 (w), 999 (w), 983 (w), 955 (m), 935 (w), 915 (w), 895 (w), 867 (m), 831 (w), 819 (w), 790 (vs), 766 (m), 746 (m), 723 (w), 680 (s). Anal. Calcd (%) (C_64_H_52_EuF_12_NO_8_, 1343.06 g mol^–1^): C, 57.24; H, 3.90; N, 1.04. Found: C, 57.10; H, 3.83; N, 1.13.

### (Et_4_N)­[Gd­(nta)_4_]

FTIR (cm^–1^), Figure S1: 1613 (vs), 1570 (w), 1528 (m), 1506 (m), 1457 (s), 1429 (m), 1385 (w), 1349 (w), 1289 (vs), 1248 (w), 1179 (s), 1130 (vs), 1072 (w), 1057 (w), 1051 (w), 1017 (w), 998 (w), 984 (w), 957 (m), 936 (w), 917 (w), 895 (w), 867 (m), 831 (w), 820 (w), 789 (vs), 767 (m), 748 (m), 720 (w), 682 (s). Anal. Calcd (%) (C_64_H_52_F_12_GdNO_8_, 1348.35 g mol^–1^): C, 57.01; H, 3.89; N, 1.04. Found: C, 54.89; H, 3.37; N, 1.04.

### Preparation of the PMMA:Eu^III^ Films

The PMMA films were prepared from mixtures of commercial PMMA and the (Et_4_N)­[Eu­(nta)_4_] complex using acetone as solvent through a drop-casting approach. Initially, PMMA was completely dissolved in acetone, followed by the addition of the europium complex at different weight loadings (1, 2, 3, 4, and 5 wt % to PMMA), yielding homogeneous precursor solutions. The mixtures were maintained under continuous stirring and heating at 60 °C until the solvent volume was reduced to approximately 2 mL. Subsequently, the resulting solutions were drop-cast onto glass substrates 5 × 2 cm^2^ at room temperature and allowed to dry for 24 h under a saturated acetone atmosphere to minimize film opacity and ensure uniform formation. After complete drying, the PMMA films spontaneously detached from the glass substrate. It is important to emphasize that the use of highly soluble complexes is particularly advantageous for LSC fabrication, as it suppresses the formation of opaque regions and aggregation domains within the polymeric matrix, thereby contributing to improved optical quality and light-guiding properties. The detailed composition of each film is summarized in Table S9.

### Fabrication of the LSC Based on the PMMA:2%Eu^III^ Film

The PMMA film doped with 2 wt % of the Eu^III^ complex, used for the LSC measurements, was prepared and deposited onto a glass substrate with a surface area of 5 × 5 cm^2^, ensuring uniform film formation over the entire substrate. Briefly, the LSC films were deposited onto glass substrates with dimensions of 5 × 5 cm^2^. After solvent evaporation and complete drying, the final thickness of the prototypes was measured as 0.23 cm, resulting in overall dimensions of 5 × 5 × 0.23 cm^3^. The silicon photovoltaic cell employed in the measurements had dimensions of 3 × 2.5 cm^2^. However, only 2.5 cm of one edge of the LSC was optically coupled to the PV cell. Therefore, the effective coupling area between the LSC edge and the PV cell was 0.58 cm^2^ (2.5 × 0.23 cm^2^). Considering the illuminated top surface area of the LSC (25 cm^2^) and the effective PV coupling area, the geometric factor (G) was calculated to be 43.5.

## Results and Discussion

### Synthesis and General Characterization of the (Et_4_N)­[Ln­(nta)_4_] Complexes

The reasoning behind the choice of the (Et_4_N)­[Eu­(nta)_4_] complex is simple: tetrakis complexes avoid water molecules coordinated to Eu^III^, reducing deactivation through vibronic coupling with OH oscillators.^26–28^ Moreover, the conjugated structure of nta^–^ extends the absorption into the near-UV-to-blue spectral region, enabling luminescence under sunlight exposure. The absence of coordinated water molecules was confirmed by thermogravimetric analysis, which also shows that the complex remains thermally stable up to 266 °C (Figure S2). One should highlight that the Gd^III^ complex was synthesized solely as a spectroscopic reference to determine the triplet and single state energies of the complex, which play a significant role in the ligand-to-Ln^III^ energy transfer dynamics.

The structure of the Eu^III^ complex was confirmed by single-crystal X-ray diffraction (SC-XRD); the experimental conditions and refinement parameters are summarized in Table S1. The crystal structure in Figure 2A reveals a mononuclear species in which Eu^III^ is coordinated by four bidentate nta^–^ ligands, generating a negatively charged [Eu­(nta)_4_]^−^ anion whose charge is compensated by the Et_4_N^+^ counterion. The complex exhibits a coordination number of eight, and no solvent molecules are present in the lattice. SHAPE analysis (Table S2) indicates that the first coordination sphere adopts a distorted *D*_*2d*_ geometry (triangular dodecahedron), Figure 2A, which is common for Ln^III^ complexes with a similar coordination environment.^29^ The Eu–O bond distances are within the 2.33–2.45 Å range (Table S3) and the bite angles of the nta^–^ ligands are between 70.38–71.83° (Table S4). The inter- and intramolecular interactions were studied by means of Hirshfeld surfaces (Figure 2B). As expected, stronger contacts are found close to the hydrogens of the naphthyl group and the fluorine atoms of CF_3_ in the nta^–^ ligand, characteristic of moderate H-bonds. Moreover, strong contacts are detected nearby the hydrogen atoms of the counterion, which is also involved in intermolecular H-bonds. The full description of the inter/intramolecular interactions as well as packing diagram is provided in Supplementary note S3.

**Figure 2:**
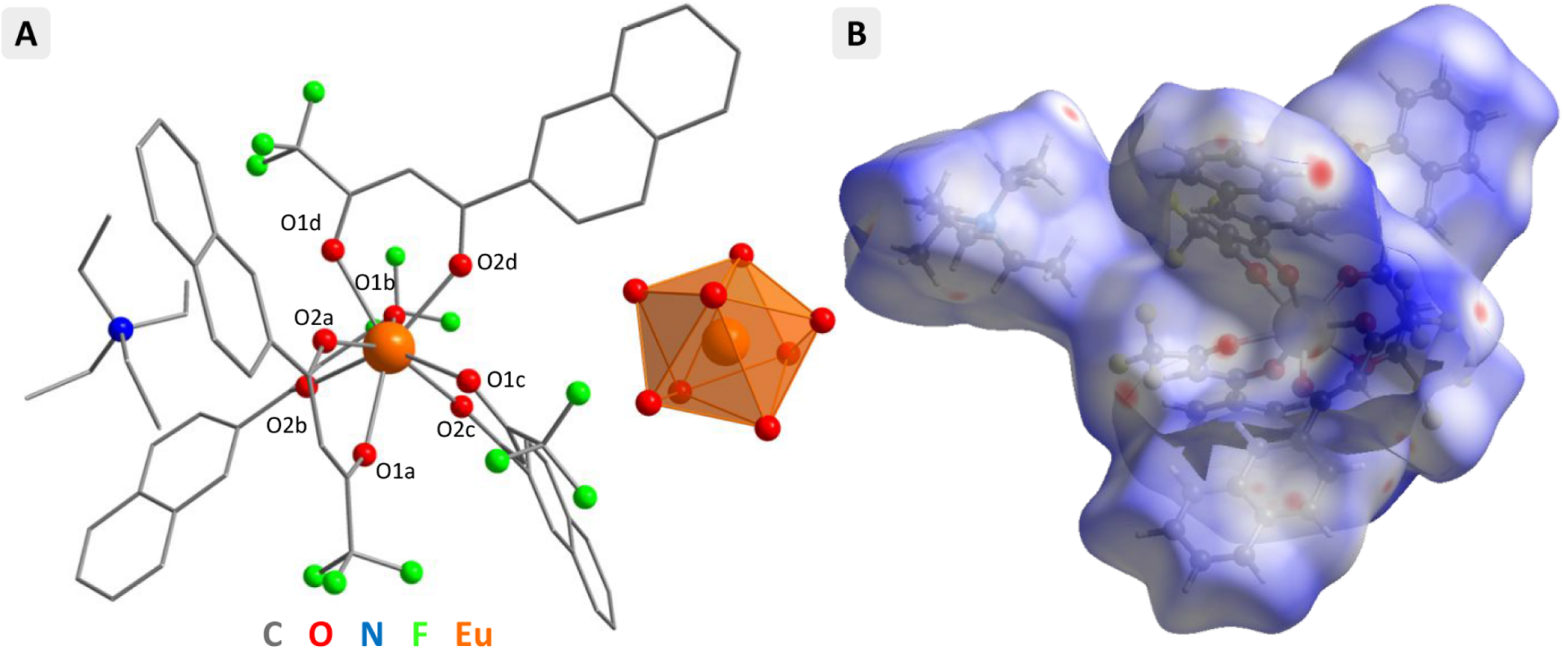
(A) Partially labeled molecular structure of (Et_4_N)­[Eu­(nta)_4_] determined from SC-XRD; hydrogen atoms have been omitted for clarity. The insert compares an idealized (orange) triangular dodecahedron (D_2d_ point group) to the actual EuO_8_ polyhedron. (B) Hirshfeld surface of (Et_4_N)­[Eu­(nta)_4_] mapped over *d_norm_* and shape index, *S*. In the *d_norm_* HS, a red–blue–white color scheme was used, whereas red regions represent closer contacts, blue regions represent longer ones, and white regions represent the distance of contacts which is exactly equal to the *vdW* separation.

To ensure that the Eu^III^ and Gd^III^ complexes adopt the same structure at 298 K, powder X-ray diffraction (PXRD) analyses were collected for the ground crystals. The experimental data (Figure S8) match the PXRD pattern simulated from SC-XRD data obtained for (Et_4_N)­[Eu­(nta)_4_], indicating that the samples retain their crystallinity after grinding and that the Eu^III^ and Gd^III^ species are indeed isostructural.

To provide a clear picture of the optical features of the Eu^III^ complex, the photoluminescence properties were investigated by means of steady-state and time-resolved spectroscopies. The excitation spectrum of (Et_4_N)­[Eu­(nta)_4_] recorded at 300 K (Figure S9A) displays the expected Eu^III^ intraconfigurational 4*f*–4*f* transitions,^30–32^ along with a broad band between 250 and 350 nm assigned to the ligand-centered S_0_ → S_n_ transitions. Furthermore, the significantly higher intensity of the S_0_ → S_1_ band relative to the Eu^III^ 4*f*–4*f* bands (Figure S9A) indicates that ligand-to-Eu^III^ energy transfer dominates the excitation dynamics compared with direct 4*f*–4*f* excitation. The T_1_ and S_1_ state energies of the complex were determined as 19,193 ± 18 cm^–1^ and 26,095 cm^–1^ by using the (Et_4_N)­[Gd­(nta)_4_] complex as spectroscopic reference, as further discussed in Supplementary note S5.

The emission spectrum of the (Et_4_N)­[Eu­(nta)_4_] complex was recorded at 300 K under ligand excitation (Figure S9B). The typical Eu^III^ narrow emission bands assigned to the ^5^D_0_ → ^7^F_0–4_ transitions are detected, rendering its characteristic red luminescence.^33^ The ^5^D_0_ excited-level lifetime of Eu^III^ was recorded from the luminescence decay curve at 300 K (Figure S12) and found to be 0.277 ms. Using this value together with the corresponding emission spectrum at 300 K, the intrinsic emission quantum yield $\left(Q_{Eu}^{Eu}\right)$ was determined to be 53%. This value is in close agreement with the overall emission quantum yield $\left(Q_{Eu}^{L}\right)$ obtained using an integrating sphere, which was 50 ± 5%. The close correspondence between these values indicates a sensitization efficiency $\left(\eta =Q_{Eu}^{L}/Q_{Eu}^{Eu}\right)$ approaching 94%, ensuring an efficient ligand-to-Eu^III^ energy transfer. Further details regarding the equations employed to determine the emission quantum yields are provided in Supplementary Note S6.

To gain insight into the energy transfer processes in the (Et_4_N)­[Eu­(nta)_4_] complex, the *JOYSpectra platform* ^34,35^ was used to calculate the energy transfer rates. The full description of the computational procedure and details about energy transfer rates are provided in Supplementary Note S7. The methodology was validated by comparing the theoretically obtained parameters with the corresponding experimental data (Table S6). The calculations used structural data obtained from SC-XRD, the experimental intensity parameters (Ω_2_ and Ω_4_) extracted from the emission spectrum (Figure S9B), the ^5^D_0_ level lifetime of the (Et_4_N)­[Eu­(nta)_4_] complex measured at 300 K, and the S_1_ and T_1_ state energies determined from the Gd^III^ complex. From these calculations, it was evidenced that the exchange-mechanism (*W*_*ex*_) dominates energy transfer from both the singlet and triplet states, corresponding to the S_1_ → (^7^F_1_ → ^5^G_2_) and T_1_ → (^7^F_1_ → ^5^D_0_) transitions (Table S8). Ligand-to-Eu^III^ back-energy-transfer from the S_1_ state $(W_{B}^{S}$ ) is lower compared to the T_1_ case, with a rate of 5.17 × 10^9^ s^–1^, whereas back-energy-transfer from the Eu^III^ (^7^F_1_ → ^5^D_0_) and (^7^F_1_ → ^5^D_1_) transitions to the T_1_ state is substantially higher, on the order of 1.52 × 10^10^ s^–1^. Overall, the photoluminescence studies indicate that the Eu^III^ complex presents an efficient near-UV-to-red downshifting conversion required for application in luminescent solar concentrators.

### Synthesis and General Characterization of the PMMA Films

For a luminescent material to function simultaneously as a LSC and as a luminescent temperature probe, several key characteristics must be fulfilled: (i) high optical transparency and homogeneity, (ii) intense luminescence, (iii) strong absorption in the near-UV spectral region to enable excitation by sunlight and subsequent re-emission within the absorption spectral range of silicon photovoltaic cells, and (iv) photo and thermal stability. To achieve these requirements, our approach involves dispersing the (Et_4_N)­[Eu­(nta)_4_] complex in the PMMA matrix to fabricate transparent luminescent films.

Polymeric films were prepared by the drop-casting method using a mixture of commercial PMMA and the synthesized (Et_4_N)­[Eu­(nta)_4_] complex dissolved in acetone. Detailed information regarding the preparation procedure is provided in Experimental section and Supplementary Note S8. The concentration of the (Et_4_N)­[Eu­(nta)_4_] complex in the PMMA matrix was varied as 1, 2, 3, 4, and 5 wt % relative to the polymer content. The film thickness ranges from 0.22 to 0.24 mm (Table S9), indicating that the dopant concentration did not influence thickness. The drop-casting method has been widely employed as a proof-of-concept approach in recent LSC-related literature^36,37^ due to its fast, simple, and low-cost implementation for screening material compositions. This strategy enables the efficient evaluation of photophysical properties and the establishment of structure–property relationships. One should note that for practical large-area application in smart window technologies, several well-established scalable alternatives are available. These include doctor-blading,^38^ spin coating^39^ for controlled thin films, slot-die coating,^40^ roll-to-roll processing,^41^ and lamination-based approaches.^42^

The films prepared by the drop-casting method are highly transparent as shown by their appearance under both artificial white light and sunlight exposure (Figure 3A). Under sunlight exposure, a red emission can be observed to the naked eye along the film edges (Figure 3A), arising from the characteristic luminescence of the Eu^III^-based complex and the waveguiding properties of the PMMA matrix. Optical transmittance measurements (Figure 3B) reveal values between 90 and 100% in the 430–800 nm spectral range, confirming the transparency of the resulting films even at higher concentrations. The dispersion of the (Et_4_N)­[Eu­(nta)_4_] complex within the PMMA matrix was confirmed by FTIR spectroscopy (Figure 3C). The FTIR spectra of the doped PMMA films display a characteristic absorption band at 1610 cm^–1^, corresponding to the ν­(C=O) vibrational mode of the complex. As expected, the relative intensity of this absorption band, compared with the PMMA carbonyl stretching band at 1718 cm^–1^, increases with higher complex loading, which is in accordance with other Eu^III^-complexes dispersed in PMMA.^22,43,44^

**Figure 3:**
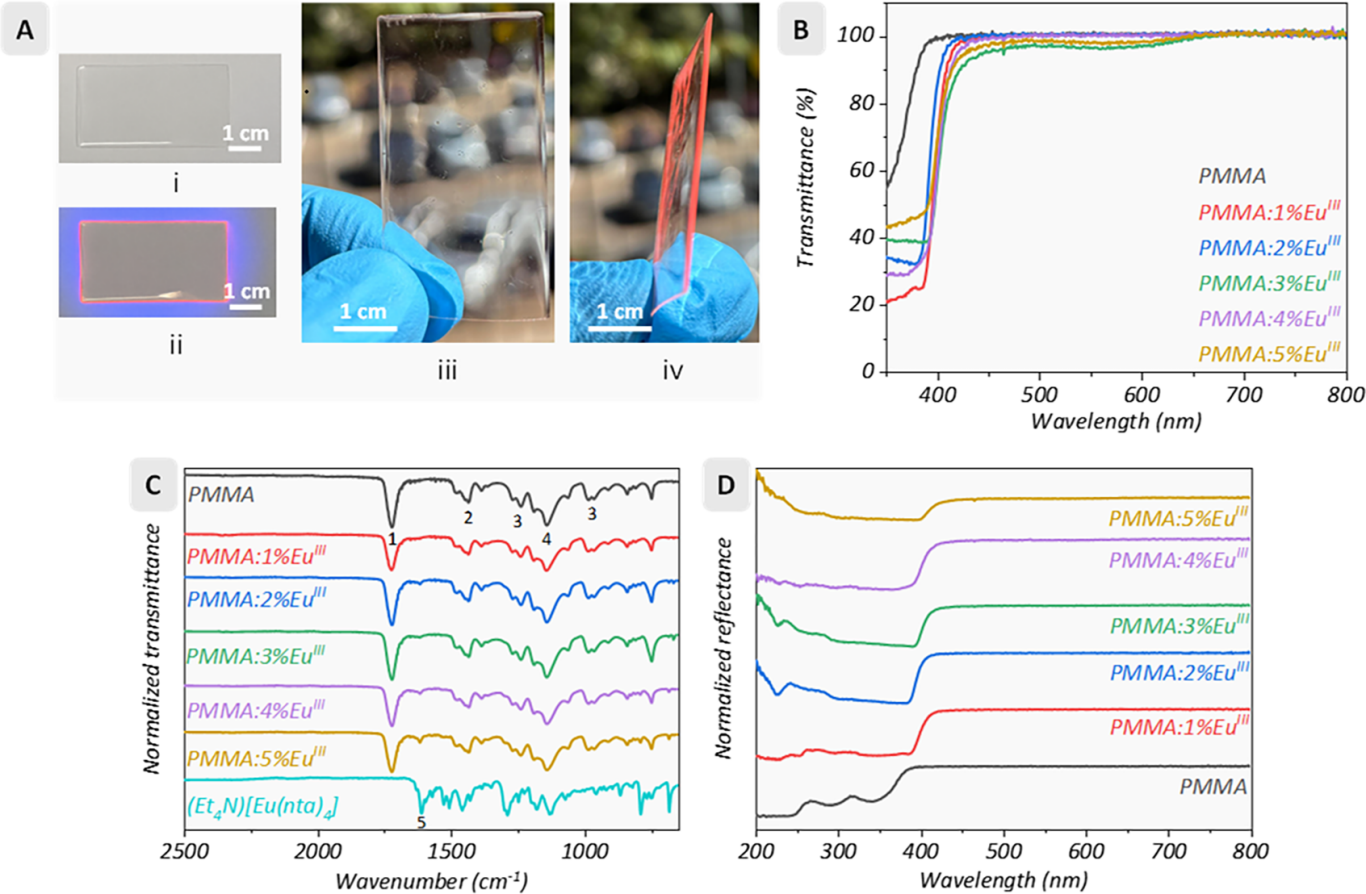
(A) Pictures of the PMMA:2%Eu^III^ film upon (i) artificial white light, (ii) UV radiation (∼390 nm), and upon sunlight exposition in (iii) front or (iv) side angles. (B) Transmittance spectra of the films. (C) FTIR of the films compared to the (Et_4_N)­[Eu­(nta)_4_] crashed crystal. Assignments: (1) ν­(C=O) of PMMA, (2) ν­(O–CH_3_) of PMMA, (3) ν­(C–O–C) of PMMA, (4) δ­(C–H), (5) ν­(C=O) of the complex. (D) Diffuse reflectance spectra of the films.

The optical absorption of the films was investigated by diffuse reflectance spectroscopy (Figure 3D). The spectra display a broad absorption band extending from 200 to 420 nm, which is assigned to the intrinsic absorption of PMMA and to ligand-centered π → π* transitions in the Eu^III^ complex, confirming that the films can absorb sunlight not efficiently captured by silicon-based photovoltaic cells. Moreover, thermogravimetric analysis shows that the PMMA:2%Eu^III^ film exhibits no significant mass loss up to 150 °C (Figure S13), ensuring its robust thermal stability within the investigated thermometry range. At temperatures above 300 °C, a pronounced weight loss is attributed to the thermal degradation of the organic ligands and subsequent decomposition of the PMMA matrix.

To evaluate the homogeneity of the films, the PMMA:5%Eu^III^ film was selected for morphological and chemical analysis, as its higher complex content facilitates detection by energy-dispersive X-ray spectroscopy (EDS). Scanning electron microscopy (SEM) images (Figure S14A) reveal a smooth and uniform surface. EDS elemental mapping (Figures S14B and S15) confirms a homogeneous distribution of Eu^III^ across the entire film, demonstrating the effective incorporation of the (Et_4_N)­[Eu­(nta)_4_] complex within the PMMA matrix.

### Photoluminescence Investigation of the PMMA Films

After synthesizing the (Et_4_N)­[Eu­(nta)_4_] complex and incorporating it into PMMA, the luminescent properties of these systems were investigated under excitation at 390 nm and sunlight exposure. The excitation spectra monitoring the Eu^III^ emission (Figure 4A) display a broad band from 250 to 410 nm, characteristic of (Et_4_N)­[Eu­(nta)_4_]. Compared with the free-standing complex, a blue shift is observed in the excitation band; nevertheless, the polymeric films remain excitable by sunlight. Among the polymeric film series, a red shift of the excitation band takes place with increasing complex content, indicating a rigidochromic effect associated with motional relaxation of the excited-state geometry, which is sensitive to the rigidity of the surrounding matrix.^45^ Additionally, π–π stacking interactions involving aromatic moieties of the nta^–^ ligands may become more pronounced at higher complex loadings, further contributing to the observed shift in the excitation band.^46^

**Figure 4:**
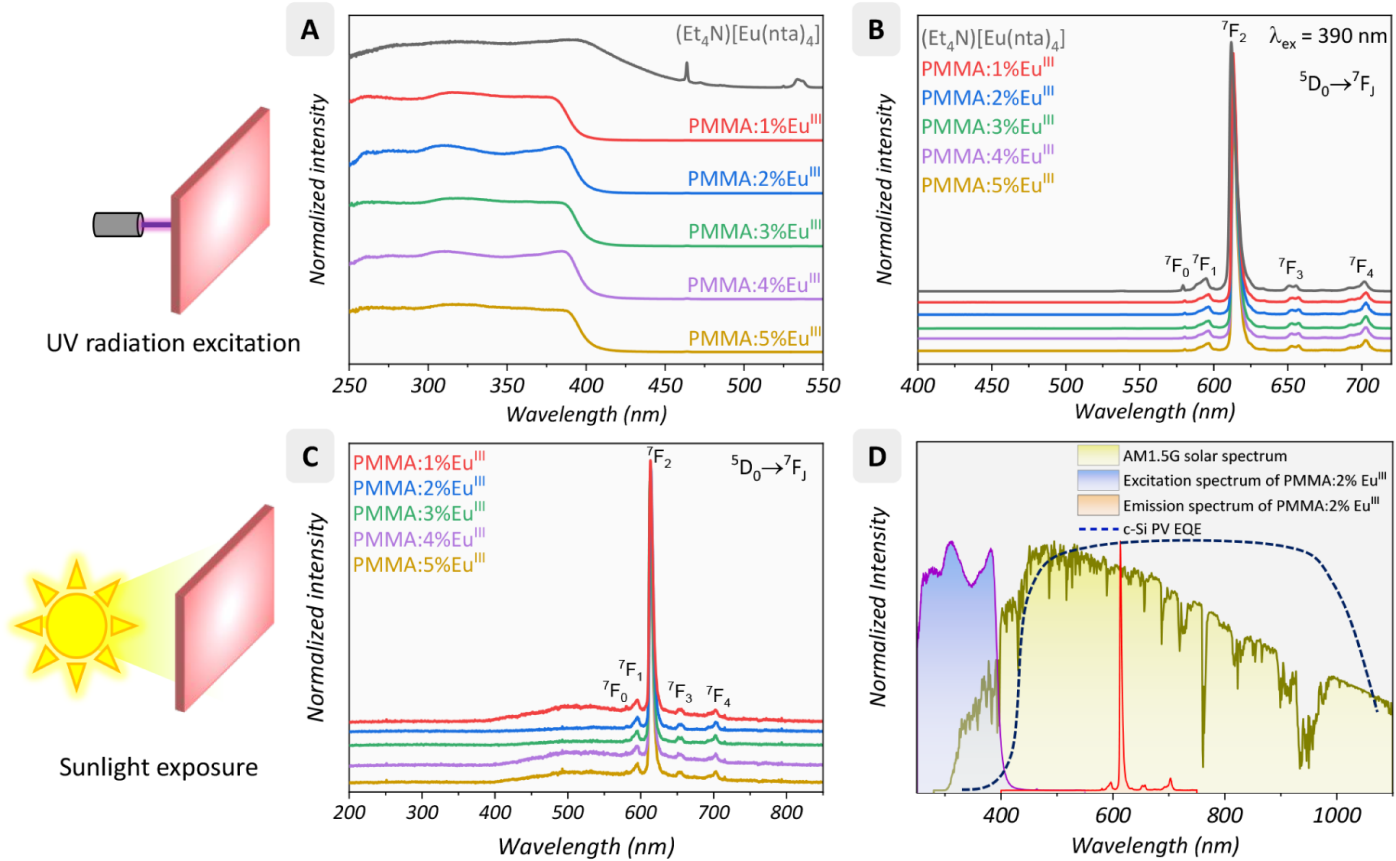
(A) Excitation spectra (300 K) of the films monitoring the Eu^III^ emission at 613 nm. (B) Emission spectra (300 K) monitored upon 390 nm excitation. (C) Emission spectra (300 K) acquired by sunlight exposure; a broad band peaking at about 510 nm is assigned to the sunlight transmitted by the optical fiber to the CCD detector. (D) AM1.5G solar spectrum (yellow), excitation spectrum of PMMA:2%Eu^III^ (blue), emission spectrum of PMMA:2%Eu^III^ (red), and c-Si PV EQE (dark blue dashed line).

Under 390 nm excitation, all films exhibit only the characteristic Eu^III^ emission peaks of (Et_4_N)­[Eu­(nta)_4_], corresponding to the Eu^III 5^D_0_ → ^7^F_J_ (J = 0–4) transitions (Figure 4B). The same behavior was observed when the emission spectra are recorded under sunlight exposure using an optical fiber to collect the luminescent signal (Figure 4C). These results suggest that the films can harvest sunlight and re-emit it within the absorption region of silicon photovoltaic (c-Si PV) cells. Figure 4D presents the spectral overlap between the solar irradiance spectrum (AM1.5G), the external quantum efficiency (EQE) curve of Silicon photovoltaic (c-Si PV) cells, as well as the excitation and emission spectra of the PMMA:2%Eu^III^ film. This comparison shows that the film efficiently absorbs in the near-UV spectral region, where the solar cell does not effectively absorb sunlight. Moreover, the emission spectrum of PMMA:2%Eu^III^ matches the maximum EQE of c-Si PV cells.

The luminescence efficiency was assessed through the photophysical properties of the films, namely the ^5^D_0_ emitting-level lifetime (τ), intrinsic emission quantum yield $\left(Q_{Eu}^{Eu}\right)$ , and overall emission quantum yield $\left(Q_{Eu}^{L}\right)$ , as described in Supplementary Note S6. Time-resolved spectroscopy (Figure S16) reveals that after dispersion of the complex in the film, the ^5^D_0_ level lifetime becomes longer (Table 1); on the other hand, the nonradiative decay rate (A_nrad_) decreases after incorporation in the films (Table 1). In the crystalline state, cross-relaxation between adjacent Eu^III^ ions can occur depending on the Eu–Eu distance, enhancing nonradiative processes in the presence of lattice defects. In the films, however, this process is suppressed by dilution effects, resulting in reduced nonradiative decay rates.^47^ Notably, the Eu^III 5^D_0_ level lifetime remains statistically unchanged among the films, suggesting that concentration barely affects the dynamics of the ^5^D_0_ level. This is reflected in the nearly identical $Q_{Eu}^{Eu}$ values (Table 1).

**Table 1:** Photophysical Parameters of the Films Compared to (Et_4_N)­[Eu­(nta)_4_] as Crystal^a^

Sample	τ (ms)	A_rad_ (s^–1^)	A_nrad_ (s^–1^)	$Q_{Eu}^{Eu}\left(\%\right)$	$Q_{Eu}^{L}\left(\%\right)$	η (%)
Et_4_N[Eu(nta)_4_]	0.277 ± 0.01	2089	1511	58	50 ± 5	86
PMMA:1%Eu	0.431 ± 0.01	1127	1194	49	42 ± 4	86
PMMA:2%Eu	0.437 ± 0.01	1190	1198	52	49 ± 5	94
PMMA:3%Eu	0.446 ± 0.01	1146	1096	51	50 ± 5	98
PMMA:4%Eu	0.438 ± 0.01	1134	1148	50	50 ± 5	100
PMMA:5%Eu	0.435 ± 0.01	1137	1162	49	46 ± 5	94

a The Reported Greatnesses are the Eu^III^ ^5^D_0_ Level Lifetime (τ), Radiative (A_rad_) and Non-Radiative (A_nrad_) Decay Rates from the Eu^III^ ^5^D_0_ Emitting Level, Eu^III^ Intrinsic Emission Quantum Yield $\left(Q_{Eu}^{Eu}\right)$ , Eu^III^ Overall Emission Quantum Yield $\left(Q_{Eu}^{L}\right)$ , and Sensitization Efficiency (η).

Incorporation of the complex into the polymer did not influence the overall emission quantum yield $\left(Q_{Eu}^{L}\right)$ which remains between 42–50% (Table 1). The $Q_{Eu}^{Eu}$ is statistically identical to $Q_{Eu}^{L}$ , indicating a ligand-to-metal sensitization efficiency close to or exceeding 90% in all the cases (Table 1). Since the photophysical properties are independent of concentration of complex, the PMMA:2%Eu^III^ film was selected for subsequent experiments due to its low complex content.

### Stability Tests of PMMA:2%Eu^III^ Film

The stability of the PMMA:2%Eu^III^ film is essential for both LSC and luminescence thermometry applications, since prolonged exposure to sunlight, temperature variations, and humidity can affect the emission properties and compromise device performance. The photostability of the PMMA:2%Eu^III^ film was evaluated by monitoring the Eu^III^ emission intensity upon continuous 390 nm excitation for 2 h (Figure S17) under different optical power density irradiation conditions. Within the investigated range, no linear relationship between emission intensity changes and irradiation power density was observed. Even under an irradiance of 9.198 W m^–2^, the emission intensity decreased by only 7% relative to its initial value. Such behavior is commonly reported for β-diketonate Eu^III^ complexes in both film and powder forms and may be associated with photodegradation processes or with changes in the packing arrangement of the complexes within the PMMA matrix, particularly at the film surface.^48,49^ The latter hypothesis is likely more relevant in this case, since the ^5^D_0_ level lifetime remained unchanged after 3 h of UV (390 nm) exposure at an irradiance of 9.198 W m^–2^ (Figure S17B), reinforcing the stability of the material under prolonged UV irradiation. In the case of photodegradation, slight changes in the ^5^D_0_ lifetime or the appearance of an additional decay component would be expected in the time-resolved measurements. It is also important to note that UV optical power density employed in the photostability tests is comparable to or higher than most of the natural UV irradiation values measured throughout the day during the LSC tests (Tables S10 and S11), indicating that natural sunlight is not expected to induce significant photodegradation in the material. Moreover, to collect the ^5^D_0_ lifetime, a pulsed lamp is employed in short acquisition times (1–10 s), which should not induce photodegradation.

Additionally, a luminescent material intended for LSC and thermometric applications must maintain high performance over extended periods and under harsh environmental conditions. To simulate these conditions, three different accelerated aging tests were performed. First, the ^5^D_0_ level lifetime was measured before and after heating the PMMA films at 60 °C for 24 h (Figures S18A and S18B). A second test was carried out under the same thermal conditions, but with the films immersed in water to simulate a high-humidity environment (Figure S18C and S18D). Finally, a third test was conducted by measuring the emission lifetime after daily exposure to solar irradiation over five consecutive days, aiming to reproduce real outdoor operating conditions (Figure S19). In all cases, no significant changes in the emission lifetime values were observed, demonstrating that the produced films maintain their luminescent performance under thermal stress, humidity exposure, and prolonged solar irradiation.

### PMMA:2%Eu^III^ Performance as LSC for Smart Window Application

To evaluate the performance as LSC, a glass substrate was coated with the PMMA:2%Eu^III^ film to enhance the effective edge area (Figure 5A), as described in Experimental Section. For that, the PMMA:2%Eu^III^ composition was drop-casted onto the glass substrate and allowed to dry, ensuring firm adhesion to the surface. This procedure increases the accessible film area and facilitates efficient current collection when the film is placed in contact with the silicon cell. The LSC assays were performed by monitoring the current generated by a commercial silicon cell coupled with the glass coated with the film, under direct sunlight exposure (Figure 5B). The current was collected hour by hour between 8 AM and 4 PM in São Paulo city and the values were compared with those obtained when the same cell was coupled only to the glass substrate or the substrate coated with the undoped PMMA film. To evaluate the thickness uniformity of the prepared samples, multiple measurements were performed at different positions across the coated glass substrates. The thickness of the glass substrate coated with the undoped PMMA film was 2.29 ± 0.02 mm, while that of the glass substrate coated with PMMA:2%Eu^III^ was 2.29 ± 0.03 mm. In comparison, the uncoated glass substrate was analyzed through nine independent measurements, all of which consistently presented a thickness of 2.19 mm. The absorbance of the three glass substrates, without any coating, was measured to confirm their identical optical behavior (Figure S20), ensuring comparability.

**Figure 5:**
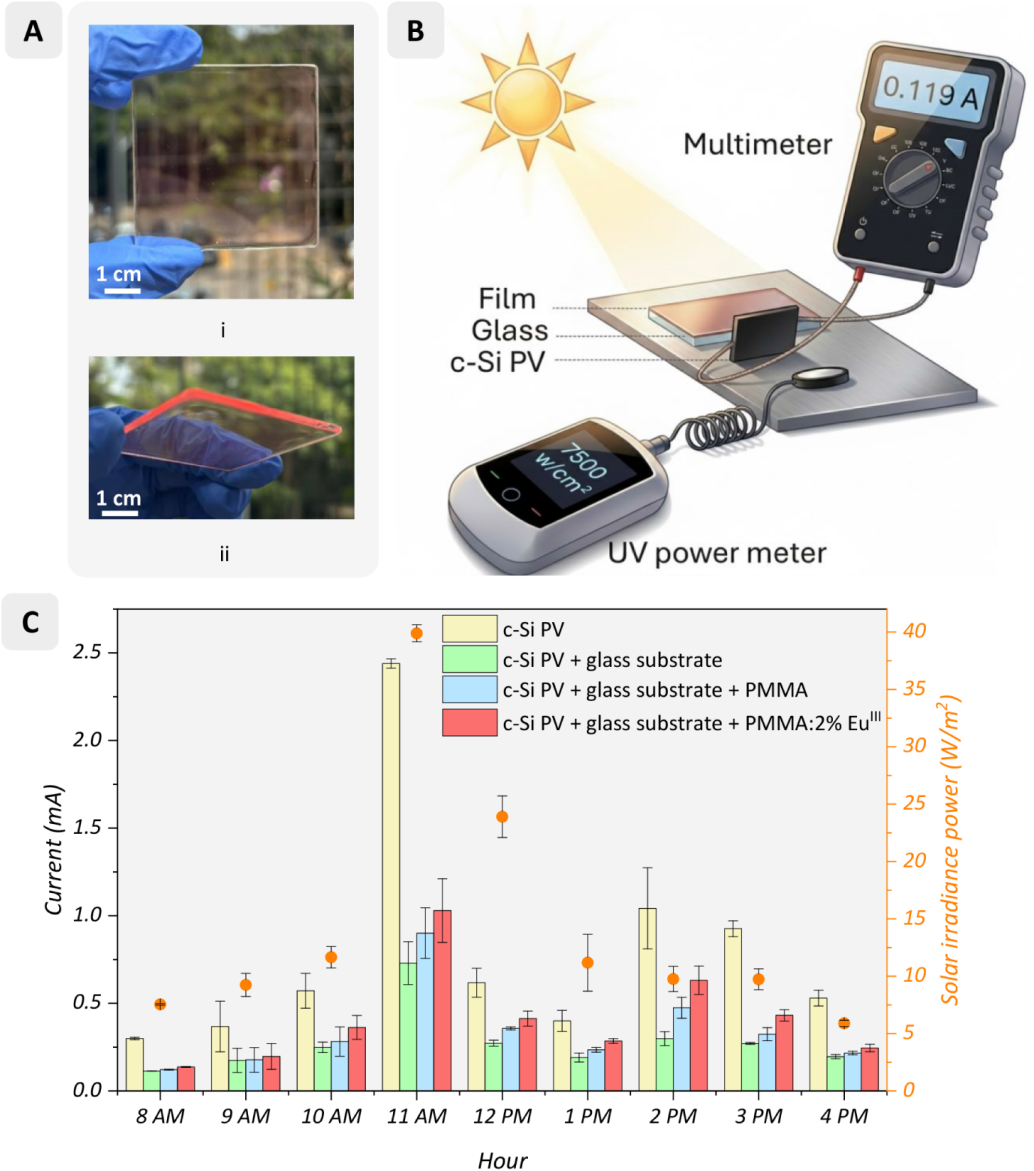
(A) Pictures of glass substrate coated with PMMA:2%Eu^III^: (i) front and (ii) side angles. (B) Schematic setup to measure the current generated by a silicon cell (c-Si PV) in contact with a glass substrate coated with the PMMA films. (C) Current generated by the bare c-Si PV (yellow), c-Si PV with a glass substrate (green), c-Si PV with a glass substrate coated with PMMA (blue), and c-Si PV with a glass substrate coated with PMMA:2%Eu^III^ (red) measured between 8:00 AM and 4:00 PM, along with the corresponding solar irradiance power at each hour.

A direct comparison between the current generated by the bare c-Si PV cell and that obtained when the c-Si PV cell was coupled to the uncoated glass substrate showed a decrease in current in all analyzed periods (Figure 5C). This reduction in photovoltaic performance is likely due to attenuation of the incident photon flux reaching the c-Si PV cell, since the glass introduces a physical barrier on the PV.^40^ However, for applications such as smart windows, the presence of a glass layer is unavoidable. To access the LSC performance, the most meaningful comparison is between the c-Si PV cell in contact with the uncoated glass substrate, and the glass coated with the undoped PMMA or the PMMA:2%Eu^III^ films. This comparison reveals larger current generated upon incorporation of the luminescent layer throughout the entire analyzed period (Figure 5C and Tables S10 and S11). The enhancement demonstrates that the PMMA:2%Eu^III^ film effectively functions as an LSC, improving the photovoltaic performance of the c-Si PV cell in a smart-window configuration. One should note that when the undoped PMMA film was employed, a modest improvement was observed relative to the uncoated glass substrate. This is not surprising considering the intrinsic waveguiding properties of the PMMA matrix, directing the light to the film edges.

Another way to evaluate the electrical performance of the LSC architectures is by the optical conversion efficiency (η_opt_) defined according to eq 1,^46,50^ where P_out_ represents the power emitted from the edge of the LSC, and P_in_ is the incident power entering through the top surface of the LSC. I_LSC_ corresponds to the short-circuit current of the silicon solar cell coupled to the LSC, whereas I_SC_ is the short-circuit current of the solar cell directly illuminated by the solar irradiance. G is the geometrical factor, defined as the ratio between the top-surface area and the edge area of the LSC. Here, the G factor was calculated to be 43.5. The experimental optical efficiency (η_opt_) was determined for four different time periods. The results show a performance of 3.4% during periods of higher solar irradiance and values close to 2.8% during periods of lower solar irradiance (Table 2) for the glass substrate coated with PMMA:2%Eu^III^. One should note that part of the photovoltaic contribution arises from the PMMA matrix itself, as the η_opt_ of the LSC increases by approximately 2.7% under higher solar irradiance and to values close to 2.4% under lower solar irradiance when undoped PMMA is employed. Thus, the contribution of the Eu^III^ complex in the PMMA:2%Eu^III^ (η_opt(c‑Si PV+glass substrate PMMA:2%Eu)_ – η_opt(c‑Si PV+glass substrate PMMA)_) lies between 0.4 and 0.8%. These results are lower but consistent with previously reported values for Eu^III^-based LSC systems, for which contributions of approximately 1.46% have been described in the literature.^46^

1 $$\eta_{opt}=\frac{P_{out}}{P_{in}}=\frac{I_{LSC}}{I_{SC}G}$$

**Table 2:** Current Data Obtained from LSC Measurements^a^

	**Irradiance** **(W/m** ^**2**^)							
	8 AM		10 AM		1 PM		4 PM	
	7.565 ± 0.063		11.647 ± 0.932		11.176 ± 2.476		5.886 ± 0.266	
Sample	Current (mA)	η_opt_ (%)	Current (mA)	η_opt_ (%)	Current (mA)	η_opt_ (%)	Current (mA)	η_opt_ (%)
c-Si PV + glass substrate	0.114	-	0.248	-	0.191	-	0.195	-
c-Si PV + glass substrate PMMA	0.121	2.4	0.282	2.6	0.234	2.7	0.216	2.5
**c-Si PV + glass substrate PMMA:2%Eu** ^**III**^	0.137	2.8	0.362	3.4	0.285	3.4	0.245	2.9
**Contribution of** **PMMA:2%Eu** ^**III**^	-	0.4	-	0.8	-	0.7	-	0.4

a The optical efficiency (η_opt_) was determined at four distinct time intervals throughout the day corresponding to different solar irradiance levels.

The optical conversion efficiency (η_opt_) obtained for the PMMA:2%Eu^III^ system (∼3.4%) falls within the range of reported values for thin-film and dye/quantum-dot-based LSC architectures operating under comparable geometrical configurations.^51–53^ As summarized in Table S12, conventional organic dye-based systems such as DCJTB, Pt­(TPBP)^54^ typically exhibit efficiencies in the range of 4–5%, while more optimized systems based on highly doped Eu^III^ complexes (e.g., PMMA:80% [Eu­(tta)_4_]­tpp)^46^ or quantum-dot-based LSCs can reach values between ∼5% and 10%, while nanostructured systems (e.g., CdSe/CdS QDs or carbon quantum dots)^55–57^ reach up to ∼10–16% under optimized conditions. Exceptional architectures based on waveguide engineering (e.g., hollow-core cylindrical devices)^58^ may report significantly higher values (>70%), although these rely on highly specialized geometries that are not directly comparable to planar smart-window configurations.

### Lifetime Thermometry of PMMA:2%Eu^III^ Film

To assess the suitability of PMMA:2%Eu^III^ for temperature monitoring, both the emission spectra and the ^5^D_0_ emitting-level lifetime were recorded as a function of temperature from 77 to 420 K. The normalized emission profiles remain essentially invariant over the entire temperature range investigated (Figure 6A), resulting in approximately constant intensity ratio between the ^5^D_0_ → ^7^F_2_ and ^5^D_0_ → ^7^F_1_ bands (Figure S21). This invariance rules out the feasibility of ratiometric luminescence thermometry in this system. As shown in Figure 6B and C, the Eu^III 5^D_0_ level lifetime is temperature-independent up to 280 K, followed by a progressive decrease up to 420 K. This behavior is consistent with thermally activated nonradiative deactivation described by the Mott–Seitz model (eq 2). In this model, τ_0_ = 1.93 ± 0.01 ms represents the radiative lifetime at 0 K, K = 3.1 ± 5.4 × 10^9^ s^–1^ is the pre-exponential factor, *K*_B_ is the Boltzmann constant, and ΔE = 5344 ± 443 cm^–1^ is the activation energy associated with thermal quenching.

2 $$\tau =\frac{1}{\tau_{0}^{-1}+k\exp\left(-\frac{\Delta E}{K_{B}T}\right)}$$

**Figure 6:**
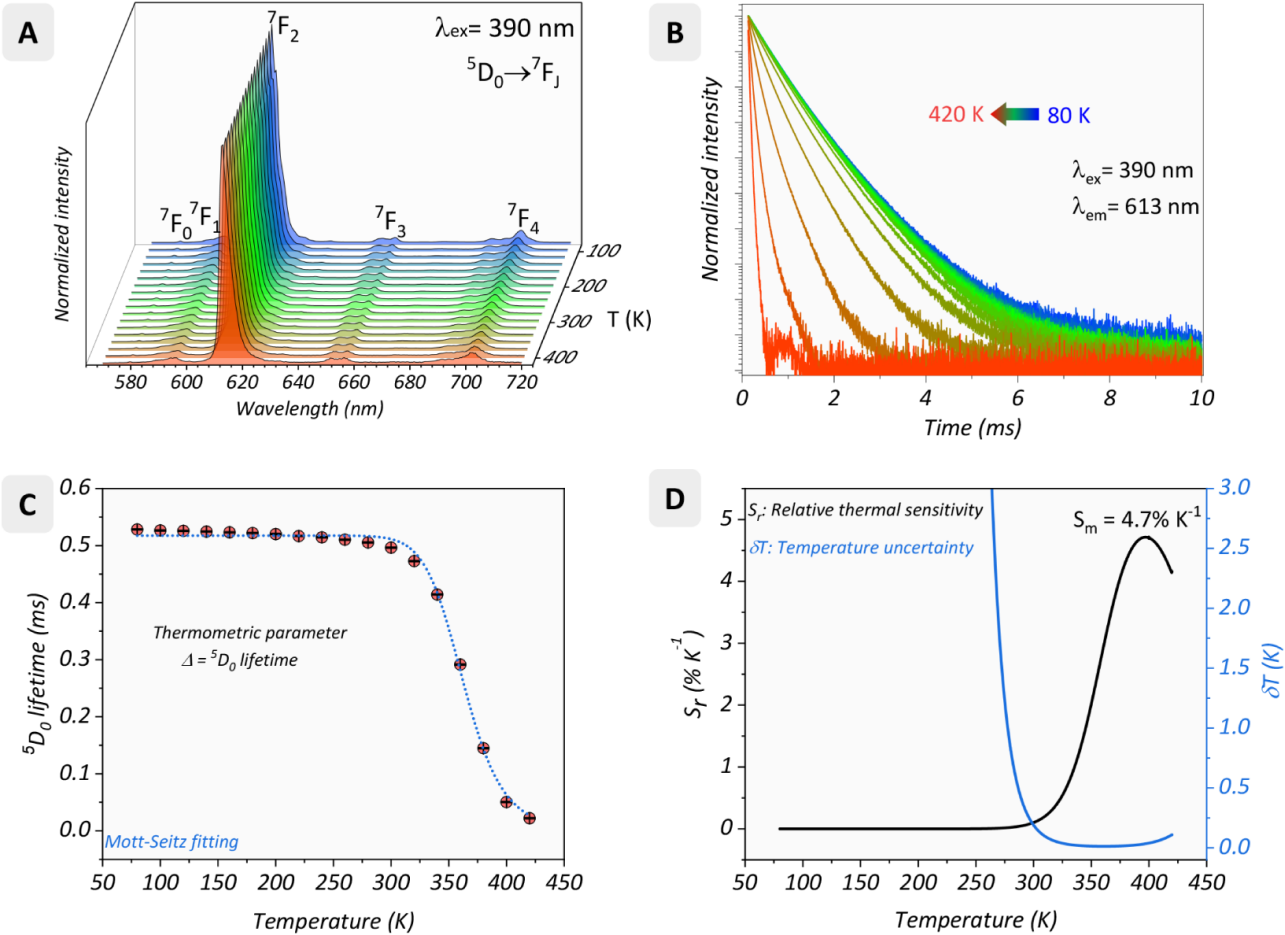
(A) Temperature-dependent (77 K–420 K, thermal rate of 10 K min^–1^) emission spectra (λ_exc_ = 390 nm) for the PMMA:2%Eu film. (B) Temperature-dependent emission decay curves (77 K–420 K, thermal rate of 10 K min^–1^) for the PMMA:2%Eu film (λ_ex_ = 390 nm and λ_em_ = 613 nm). (C) Dependency of the ^5^D_0_ level lifetime on the temperature and data best fitting to a Mott–Seitz function; the ^5^D_0_ level lifetime was used as the thermometric parameter (Δ) for the luminescence thermometry assay. (D) Temperature dependency of the relative thermal sensitivity (S_r_) and temperature uncertainty (δT) calculated from the dependency of the thermometric parameter on temperature. For clarity of visualization due to scale limitations, temperature uncertainty was only displayed for values below 3.0 K.

Before evaluating the thermometric performance of the PMMA:2%Eu^III^ film, the mechanism responsible for the thermal quenching of the luminescence was thoroughly investigated. To assess whether the changes in the ^5^D_0_ lifetime could arise from a phase transition rather than from a photophysical process centered in the complex, differential scanning calorimetry (DSC) measurements were performed for the PMMA:2%Eu^III^ film during sequential heating and cooling cycles using a thermal rate of 10 °C min^–1^ (Figure S22). Upon heating, a glass transition is observed in the 78–96 °C range, in agreement with values reported for other PMMA-based films.^59^ Upon cooling, this transition occurs within the 74–53 °C range, revealing a thermal hysteresis behavior that is characteristic of second-order phase transitions such as glass transitions.

We recently demonstrated, through combined experimental and computational approaches, that in the [Eu_2_(bpm)­(nta)_6_] complex (bpm = 2,2′-bipyrimidine), which contains the same nta^–^ ligand, the main origin of thermal luminescence suppression is the Eu^III^ → ligand back-energy-transfer process.^66^ Experimental observations further support that this pathway is the main source of luminescence quenching rather than the glass transition in the PMMA:2%Eu^III^ film. First, since the same heating rate was employed in both the DSC (Figure S22) and thermometric measurements (Figure 6B), a change in the ^5^D_0_ lifetime would be expected near the onset of the glass transition if the phase transition controlled the luminescence behavior. However, luminescence quenching begins at approximately 300 K, whereas the glass transition starts only at 78 °C (351 K).

Considering the thermal hysteresis observed in the DSC measurements (Figure S22), a corresponding hysteresis in the temperature dependence of the ^5^D_0_ lifetime could be detected^60^ during heating and cooling cycles if the glass transition significantly affected the luminescence behavior. To verify this possibility, the temperature dependence of the ^5^D_0_ lifetime was measured during both heating and cooling cycles using the same heating rate employed in the DSC experiments (10 °C min^–1^), as shown in Figure S23. No significant differences were observed between the heating and cooling curves, demonstrating the absence of thermal hysteresis in the thermometric response. This result confirms that the ^5^D_0_ lifetime can be reliably measured independently of the thermal cycle, validating its applicability for luminescence thermometry. Therefore, although the glass transition occurs within a similar temperature range in which the ^5^D_0_ lifetime alterations are observed, this physical process does not contribute significantly to the thermal suppression of the Eu^III^ emission.

After evaluating the mechanism behind the luminescence dependence on the temperature, the^5^D_0_ level lifetime was employed as a thermometric parameter to determine the relative thermal sensitivity (*S*_r_) of the luminescence-based thermometric assay (Figure 6D) according to eq S21, with the full derivation provided in Supplementary Note S11. As expected from the temperature-independent behavior of the ^5^D_0_ lifetime below 280 K, *S*_r_ remains essentially negligible throughout this low-temperature interval. Above this threshold, however, the lifetime begins to decrease monotonically, leading to a pronounced enhancement in thermal sensitivity and yielding a maximum relative sensitivity (*S*_r_) of 4.7% K^–1^ at 397 K. This value is remarkably high when compared with the state-of-the-art Eu^III^-based systems that exploit ^5^D_0_ level lifetime for luminescence thermometry (Table S13).^21,61–66^

Temperature uncertainties were calculated using eq S22 presented in Supplementary Note S11, resulting in minimum uncertainties of 0.77 at 280 K and 0.10 at 420 K within the low- and high-temperature regimes, respectively (Figure 6D). Although thermal quenching leads to a decrease in emission intensity and signal-to-noise ratio at higher temperatures, no significant deterioration in the luminescence decay fitting quality was observed owing to the high detector sensitivity and large emission quantum yield of the PMMA:2%Eu^III^ film, enabling reliable determination of both the relative thermal sensitivity and temperature uncertainty throughout the investigated temperature range.

The operational temperature-sensitivity regime investigated in this study lies above the average indoor temperatures typically observed in residential environments worldwide. Nevertheless, temperatures in the range of 30–60 °C (303–333 K), or even higher, are readily reached in outdoor environments such as rooftops and parking lots, where the implementation of smart window technologies is particularly relevant.^67^ Moreover, such locations are well-suited for energy storage, as solar irradiation can be relatively constant over extended periods. Within this temperature interval, the thermal sensitivity was determined to range from 0.14 to 0.89% K^–1^, which is consistent with values reported in previously studies.^23,24^

Collectively, our results show that the fabricated film demonstrates high optical transparency, strong absorption in the near-UV spectral region, luminescence upon sunlight exposition that increases the efficiency of c-Si PV cells, and luminescence thermometry capability near room temperature. Although this technology is not competitive with traditional photovoltaics in terms of absolute efficiency, it is complementary. In fact, LSC modules can be applied in scenarios where conventional silicon-based photovoltaics are unsuitable, such as smart windows, where transparent materials are required to preserve luminous flux. These features underscore the potential of this approach as a viable pathway to next-generation smart optical materials that simultaneously enable LSC functionality and high-performance luminescent temperature sensing.

## Conclusion

In this work, we demonstrated that PMMA films doped with the highly emissive (Et_4_N)­[Eu­(nta)_4_] complex constitutes an effective dual-function optical platform capable of simultaneously harvesting solar energy and providing reliable luminescent temperature sensing. The conjugated β-diketonate ligand ensures efficient sunlight absorption and near-unity sensitization efficiency, while the PMMA matrix preserves high transparency and allows homogeneous dopant dispersion. As a result, the films exhibit strong Eu^III^ red emission (absolute emission quantum yeilds of 50%), efficient waveguiding, and enhancement in current generation in a smart window prototype, validating their applicability as luminescent solar concentrators. Furthermore, temperature-dependent lifetime measurements reveal a pronounced thermally activated quenching of the Eu^III 5^D_0_ level, yielding a maximum relative thermal sensitivity of 4.7% K^–1^ at 397 K, among the highest reported for lifetime-based Eu^III^ luminescent temperature probes. These findings highlight the potential of Eu^III^-doped PMMA films as transparent, photostable, and multifunctional materials for smart-window technologies, offering a viable pathway toward energy-efficient and self-monitoring architectural systems.

## Supplementary Material







## Notes

The crystal structure has been deposited at the Cambridge Crystallographic Data Center (CCDC); CCDC number is 2532838 (CIF)
Joyspectra raw data (TXT)
Characterization and data analysis procedures (PDF)
