# Supplementary material for: Multifunctional Luminescent Solar Concentrator Integrating Optical Thermometry Based on PMMA/EuIII-Complex Films for Smart Windows
Source: ACS Appl Mater Interfaces. 2026 Jul 6;18(28):39001–13. doi: 10.1021/acsami.6c03810 (PMC13397481; doi:10.1021/acsami.6c03810)
Supplement: Supplementary file 3 [file am6c03810_si_003.pdf]

Supplementary information

Multifunctional Luminescent Solar Concentrator Integrating Optical Thermometry Based on PMMA/Eu^{III}-Complex Films for Smart Windows

Felipe Canisares,[a*] Mariana V. Corrêa,[b] Juliana Izidoro,[b] Paulo R. S. Santos,[a] João H. A. Neto,[a] Javier A. Ellena,[c] Airton G. Bispo-Jr[a],[**] Maria Claudia F. C. Felinto,[d] Oscar L. Malta,[e] Hermi F. Brito[a***]

[a] Department of Fundamental Chemistry, Institute of Chemistry, University of São Paulo (USP), São Paulo, São Paulo 05508-000, Brazil.

[b] Scientific Learning Program, Dante Alighieri School, São Paulo 01420-002, São Paulo, Brazil.

[c] São Carlos Institute of Physics, University of São Paulo (USP), São Carlos 13566-590, São Paulo, Brazil.

[d] Nuclear and Energy Research Institute, São Paulo 05508-900, São Paulo, Brazil.

[e] Federal University of Pernambuco, Recife 50670-901, Pernambuco, Brazil.

* canisares@usp.br

** airton.bispo.junior@iq.usp.br

*** hefbrito@iq.usp.br

Keywords: multifunctional material, solar concentrator, sunlight excitation, optical conversion efficiency, thermometry.

Content

Supplementary note S1 – Characterization of the $(Et_4N)[Ln(nta)_4]$ complexes 3

Supplementary note S2 – Single-crystal X-ray diffraction 7

Supplementary note S3 – Packing diagram and inter-intramolecular interactions in $(Et_4N)[Eu(nta)_4]$ as crystal 9

Supplementary note S4 – Powder X-ray diffraction of the complexes 13

Supplementary note S5 – Further spectroscopic data of the $(Et_4N)[Ln(nta)_4]$ complexes as crystals 14

Supplementary note S6 – Calculations of the experimental photophysical properties of Eu^{III} 17

Supplementary note S7 – Computational calculations of the luminescence dynamics of $(Et_4N)[Ln(nta)_4]$ complexes as crystals 18

Supplementary note S8 – Characterization of the PMMA:Eu^{III} films 23

Supplementary note S9 – Stability tests of PMMA:2%Eu^{III} film 26

Supplementary note S10 – LSC tests 29

Supplementary note S11 – Lifetime Thermometry of PMMA:2%Eu^{III} film 32

Supplementary references 34

Supplementary note S1 – Characterization of the $(Et_4N)[Ln(nta)_4]$ complexes

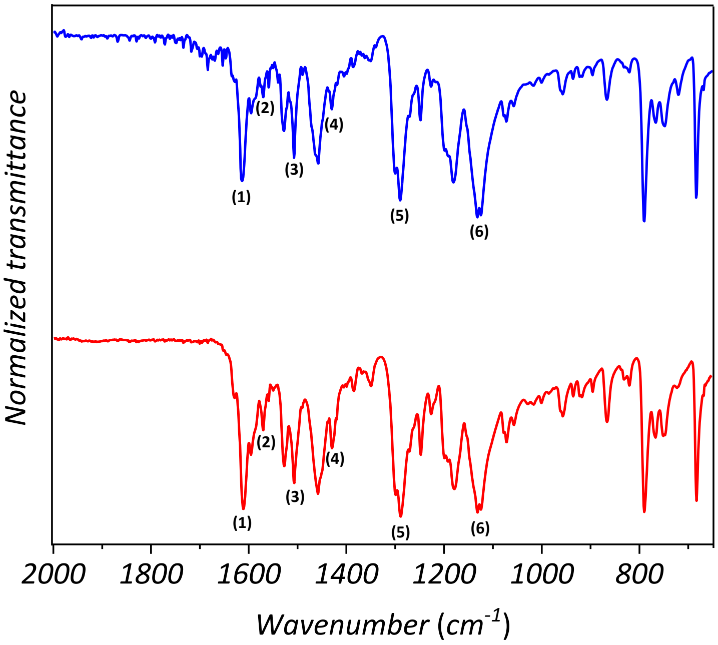

(1)	ν(C=O)
(2)	ν(C=N)
(3)	ν(C-CH_3) + ν(C=C)
(4)	ν(C=C)
(5)	ν(C-H)
(6)	ν(C-H) + ν(C-CF_3)

Figure S1. FTIR spectrum of $(Et_4N)[Eu(nta)_4]$ (red) and $(Et_4N)[Gd(nta)_4]$ (blue) crashed crystals, highlighting the assignments of the main vibrational modes.

Characterization

Fourier Transform Infrared Spectroscopy (FTIR): FTIR spectra of the complexes and films were undertaken in an Agilent Cary 600 Series FTIR Spectrophotometer (660) in the range of 4000 to 650 cm^{-1} (resolution of 2 cm^{-1}), by employing an attenuated total reflectance (ATR) accessory.

Thermogravimetry (TG): Thermogravimetric analyses of the complexes and the PMMA:2%Eu^{III} film were carried out in a TGA Q500 (TA Instruments, software version 20.13). The measurements were performed from 25 to 950 °C at a heating rate of 10 °C min^{-1} under a mixed atmosphere of air (40 mL min^{-1}) and nitrogen (60 mL min^{-1}).

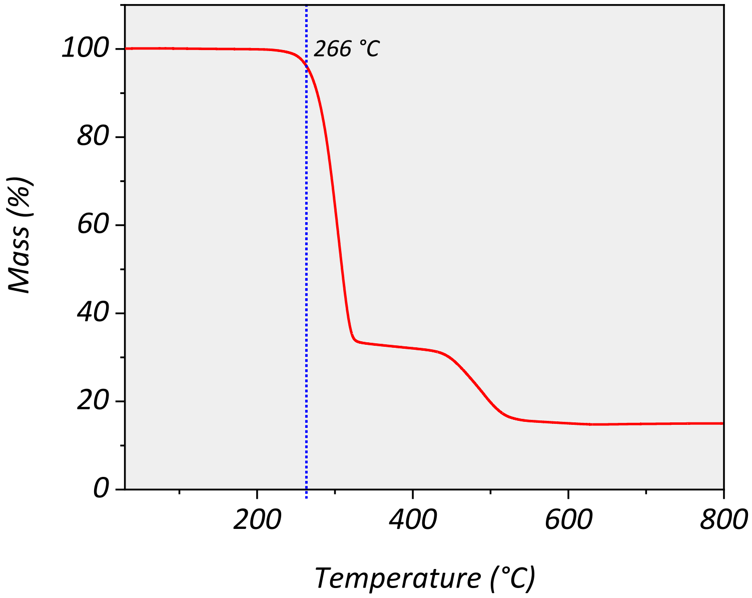

Figure S2. Thermogravimetric analysis of $(Et_4N)[Eu(nta)_4]$.

Elemental analysis (CHN): Elemental analysis was performed using a PerkinElmer 2400 Series II elemental analyzer.

Scanning electronic microscopy (SEM): SEM analyses were performed using a field-emission scanning electron microscope (FESEM, JEOL JSM-7401F) equipped with a backscattered electron detector, operating under high-vacuum conditions at an accelerating voltage of 15 kV. Semiquantitative elemental mapping was carried out by energy-dispersive X-ray spectroscopy (EDS) using the same instrument. Prior to imaging, the film surfaces were coated with a thin gold layer by DC magnetron sputtering to improve electrical conductivity.

Thickness. The thickness of the films was measured using a digital micrometer from Digimess.

Diffuse reflectance spectroscopy (DRS) and transmittance spectra: DRS and transmittance spectra were recorded using a Shimadzu UV-2600 UV–vis spectrophotometer equipped with solid-state and liquid sample holders. DRS measurements were performed using a $BaSO_4$-coated integrating sphere, with $BaSO_4$ employed as the reflectance reference. Transmittance spectra were acquired using the liquid sample holder, with the films mounted perpendicular (90°) to the optical path, and air used as the reference. For both DRS and transmittance measurements, the spectral resolution was set to 1 nm.

Photoluminescence (PL). PL measurements were performed using a Fluorolog-QM spectrofluorometer (Horiba Jobin Yvon) equipped with double-grating excitation and emission

monochromators. Excitation and emission spectra were acquired in the front-face geometry, with the excitation and emission slit widths adjusted to provide a spectral bandpass of 1 nm. For steady-state measurements, an ozone-free 450 W xenon lamp was used as the excitation source, and the emitted light was detected using a photomultiplier tube (PMT, Hamamatsu R928P) operated at 1050 V. All excitation and emission spectra were corrected for the wavelength-dependent response of the excitation source, emission monochromator optics, and photomultiplier detector. Time-resolved PL measurements were carried out using a 150 W pulsed xenon lamp coupled to a time-correlated single-photon counting (TCSPC) system. Temperature-dependent measurements were performed in the range of 100–420 K using a Lake Shore 335 temperature controller connected to a Janis VPF-800 cryostat.

Emission spectra under sunlight excitation. The emission spectra of the PMMA-doped films under sunlight irradiation were recorded using an Ocean Optics fiber optics (diameter 1 mm) connected to an Ocean Optics QE65000 spectrometer with resolution up to 1 nm.

Photodegradation of the PMMA:2%Eu^{III} film. Steady-state photoluminescence measurements were carried out using a FluoroMax Plus spectrofluorometer equipped with a vertically mounted continuous-wave (CW) 150 W ozone-free xenon arc lamp as the excitation source. The instrument employs Czerny–Turner monochromators with fully reflective optics. Emission signals were detected using an R928P photon-counting photomultiplier tube (PMT) operating in the 185–850 nm spectral range, while a reference photodiode was used to monitor fluctuations in the lamp output during measurements. The emission intensity of the PMMA:2%Eu^{III} film was continuously monitored under 390 nm excitation at different irradiance power densities (0.251, 1.161, 4.575, and 9.198 W m^{-2}) for 2 h in order to evaluate the influence of UV exposure on the photostability of the material. Additionally, the film was exposed to the highest irradiance power density (9.198 W m^{-2}) for 3 h, and the emission lifetime was measured before and after UV irradiation to investigate possible changes in the radiative and non-radiative deactivation processes induced by prolonged exposure.

Absolute emission quantum yield. PLQY measurements were performed using a Quanta Phi-2 integrating sphere coupled to the previously described Fluorolog-QM spectrofluorometer. As a reference, the empty integrating sphere coated with Spectralon® (reflectance >95%) was employed. The emission quantum yield was calculated according to Equation S1, where where N_{Em} and N_{Abs} correspond to the number of photons emitted and absorbed by the sample, respectively. I_{em} represents the emission spectrum of the sample, while I_{ex} and I_{ex}^{st} denote the excitation spectra of the incident radiation used to excite the sample and of the empty integrating sphere, respectively.

$$\Phi_L^{Ln} = \frac{N_{Emi}}{N_{Abs}} = \frac{\int_{\lambda_1}^{\lambda_2} I_{em}(\lambda)d\lambda}{\int_{\lambda_3}^{\lambda_4} I_{ex}^{st}(\lambda)d\lambda - \int_{\lambda_3}^{\lambda_4} I_{ex}(\lambda)d\lambda} \quad \text{(S1)}$$

Electrical Current Measurements under Sunlight Irradiation. The electrical current measurements were performed using a LWJ-115 LUATEK multimeter operating in the milliampere (mA) range. Current generation was achieved using a silicon photovoltaic (c-Si PV) cell with dimensions of 30 x 25 mm. The active area of the Si PV was partially masked with black tape to restrict the light-accessible region to match the thickness of the glass substrate coated with PMMA:2%Eu^{III}. This configuration ensured that only the current generated by photons waveguided to the Si PV was collected. Simultaneously, the incident solar irradiance was monitored using an LS125 UV light meter (Linshang). The data were collected in the Institute of Chemistry of University of São Paulo – Butantã Campus in São Paulo city (-23.565153368983204, -46.72584401534255) on December 16th under partly cloudy conditions with clear skies at 11:00 AM, followed by rainfall after 4:00 PM. The temperature ranged from 25.2 °C at 8:00 AM to 24.2 °C at 4:00 PM, reaching a maximum of 31.0 °C at 2:00 PM. The relative humidity varied from 75.7% at 8:00 AM to 81.8% at 4:00 PM, with a minimum of 52.5% at 3:00 PM. Atmospheric pressure remained nearly constant during data acquisition, ranging between 919 and 921 hPa.[1]

Supplementary note S2 – Single-crystal X-ray diffraction

Table S1. Crystal data and structure refinement for $(Et_4N)[Eu(nta)_4]$.

**Empirical formula**	$C_{64}H_{52}EuF_{12}NO_8$
**CCDC number**	2532838
**Empirical formula**	1343.073
**Temperature / K**	100.0(9)
**Crystal system**	Monoclinic
**Space group**	$P2_1$
**a / Å**	11.96589(11)
**b / Å**	21.67266(15)
**c / Å**	12.74180(13)
**α /°**	90
**β /°**	117.4532(12)
**γ /°**	90
**Volume /Å^3**	2932.26(6)
**Z**	2
**ρ_{calc} g cm^{-3}**	1.521
**μ / mm^{-1}**	8.482
**F(000)**	1345.9
**Crystal size / mm^3**	0.102 × 0.08 × 0.033
**Radiation**	Cu Kα (λ = 1.54184)
**2θ range for data collection/°**	9.28 to 140.12
**Index ranges**	-15 ≤ h ≤ 12, -27 ≤ k ≤ 26, -15 ≤ l ≤ 16
**Reflections collected**	63416
**Independent reflections**	10495 [R_{int} = 0.0563, R_{sigma} = 0.0382]
**Data/restraints/parameters**	10495/142/804
**Goodness-of-fit on F^2**	1.041
**Final R indexes [I>=2σ (I)]**	R_1 = 0.0357, wR_2 = 0.0893
**Final R indexes [all data]**	R_1 = 0.0377, wR_2 = 0.0902
**Largest diff. peak/hole / e Å^{-3}**	0.90/-0.48
**Flack parameter**	-0.0044(12)

Table S2. Shape analysis of the Eu^{III} coordination polyhedron in $(NEt_4)[Eu(nta)_4]$ using SHAPE 2.1.[2] Values in the table are the continuous shape measures (CShM, dimensionless) for each idealized geometry.

Idealized geometry	Short name	Point group	
**Triangular dodecahedron**	**TDD-8**	$\boldsymbol{D_{2d}}$	0.720
**Biaugmented trigonal prism**	BTPR-8	C_{2v}	1.821
**Square antiprism**	SAPR-8	D_{4d}	2.116
**Biaugmented trigonal prism J50**	JBTPR-8	C_{2v}	2.301
**Snub diphenoid J84**	JSD-8	D_{2d}	2.639
**Cube**	CU-8	*Oh*	10.405
**Triakis tetrahedron**	TT-8	*Td*	10.987
**Johnson gyrobifastigium J26**	JGBF-8	D_{2d}	11.210
**Hexagonal bipyramid**	HBPY-8	D_{6h}	15.786
**Elongated trigonal bipyramid**	ETBPY-8	D_{3h}	23.035
**Heptagonal pyramid**	HPY-8	C_{7v}	23.690
**Johnson elongated triangular bipyramid J14**	JETBPY-8	D_{3h}	28.150
**Octagon**	OP-8	D_{8h}	31.318

Table S3. Eu – O bond distances (Å) in the first coordination sphere of $(Et_4N)[Eu(nta)_4]$ crystal.

Bond	Distances / Å
Eu – O1a	2.4523(19)
Eu – O2a	2.3549(30)
Eu – O1b	2.4225(30)
Eu – O2b	2.3380(22)
Eu – O1c	2.4105(24)
Eu – O2c	2.3418(30)
Eu – O1d	2.4324(21)
Eu – O2d	2.3462(25)

Table S4. Bite angles (°) of ligands in $Et_4N[Eu(nta)_4]$ crystal.

Bond	Angle / °
O1a – Eu – O2a	70.569(88)
O1b – Eu – O2b	70.377(80)
O1c – Eu – O2c	71.826(77)
O1d – Eu – O2d	71.046(97)

Supplementary note S3 – Packing diagram and inter-intramolecular interactions in $(Et_4N)[Eu(nta)_4]$ as crystal

Inter- and intramolecular interactions in the crystal were also mapped to understand the crystal packing. The shortest Eu ··· Eu intermolecular distance is 11.67 Å, as fully viewed in the packing diagram (Figure S3). Different intramolecular H-bonds are detected between the hydrogens of the naphthyl group and the fluorines in nta^- neighbours (Figure S4 and Table S5); similar interactions are noticed between hydrogen and oxygen atoms of neighbour nta^- ligands. Intermolecular H-bonds (Figure S5 and Table S5) are also present between the fluorine or oxygen atoms with hydrogens of neighbour nta^- ligands; intermolecular H-bonds also occur between the CF_3 groups in the ligand and hydrogen atoms present in the counterion. One should note that an intermolecular F···F contact is observed between neighbouring units of the complex, with distance of 2.741 Å and angle of 150.70° (Figure S6). 2D fingerprint plots (Figure S7) reveal an overall contribution of F···H and O···H hydrogen bonds of 25.5 and 6.4%, respectively, and F···F contacts of 3.7%. Yet, the inter/intramolecular interactions are dominated by H···H (30.5%) and C···H (28.2%) contacts.

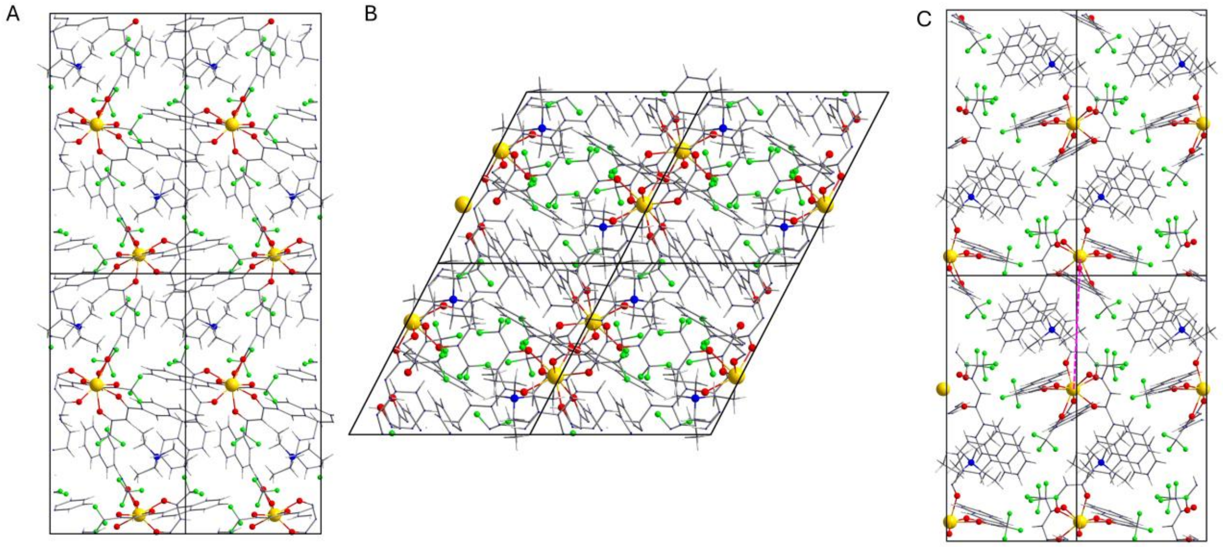

Figure S3. View of the packing arrangement along the crystallographic (A) a, (B) b, and (C) c-axis in $(Et_4N)[Eu(nta)_4]$ crystal. Pink dashed lines represent the shortest Eu · · · Eu intermolecular distance (11.68 Å). Carbon = gray, fluorine = green, nitrogen = blue, europium = yellow. Hydrogen atoms have been omitted for the sake of clarity.

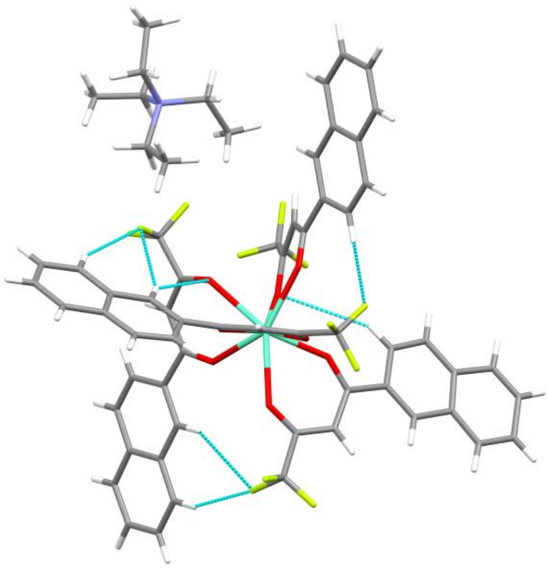

Figure S4. Intramolecular H-bonds (blue dashed lines) in the $(Et_4N)[Eu(nta)_4]$ crystal. Carbon = gray, hydrogen = white, fluorine =yellow, nitrogen = violet, europium = cyan.

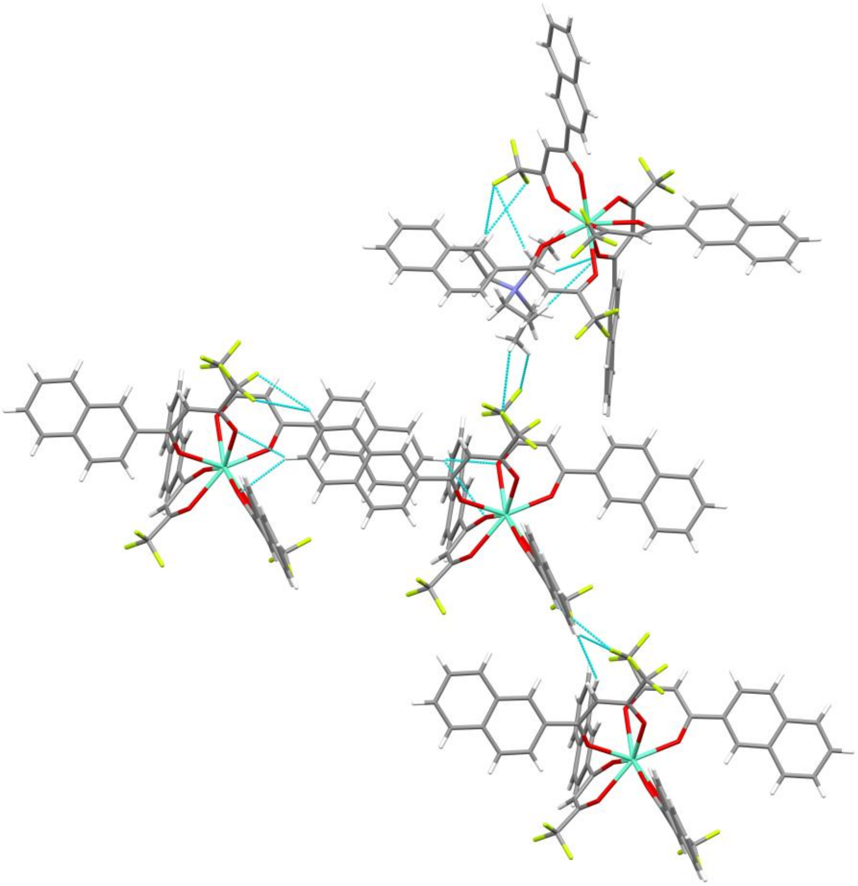

Figure S5. Representation of the intermolecular H-bonds (dashed blue lines) in $(Et_4N)[Eu(nta)_4]$ crystal. Carbon = gray, hydrogen = white, fluorine =yellow, nitrogen = violet, europium = cyan.

Table S5. Intermolecular and intramolecular H-bond distances (Å) in $(Et_4N)[Eu(nta)_4]$ crystal.

**Intramolecular H-bond / Å**		**Intermolecular H-bond / Å**	
**O1a···H14b**	3.206	**F1a···H6d**	3.108
**O1c···H5d**	3.203	**F1a···H8d**	3.147
**H6a···F2d**	3.133	**H12b···F3d**	3.037
**F2a···H12b**	3.222	**F3a···H13c**	2.765
**F2a···H14b**	3.190	**H14a···F2b**	3.113
**F2b···H14c**	2.702	**H3a···F2b**	3.042
**F2d···H8a**	3.006	**H11a···F3d**	2.957
		**O1a···H10a**	2.576
		**O2b···H10a**	3.239
		**H10c···O2d**	2.686
		**H10c···O1c**	2.633
		**H9c···F3c**	2.956
		**H9c···F2c**	3.124
		**F1c···H6b**	3.236
		**F1c···H3b**	3.000
		**F2c···H3b**	3.167
		**O2c···H7b**	2.935
		**F3a···H4b**	2.815
		**F1c···H4c**	3.206
		**F2c···H8b**	3.206
		**O2b···H1a**	2.380
		**O2b···H6g**	3.253
		**F1d···H8f**	3.070
		**F2d···H1b**	3.284
		**F2d···H8f**	2.665
		**F1b···H6e**	2.507

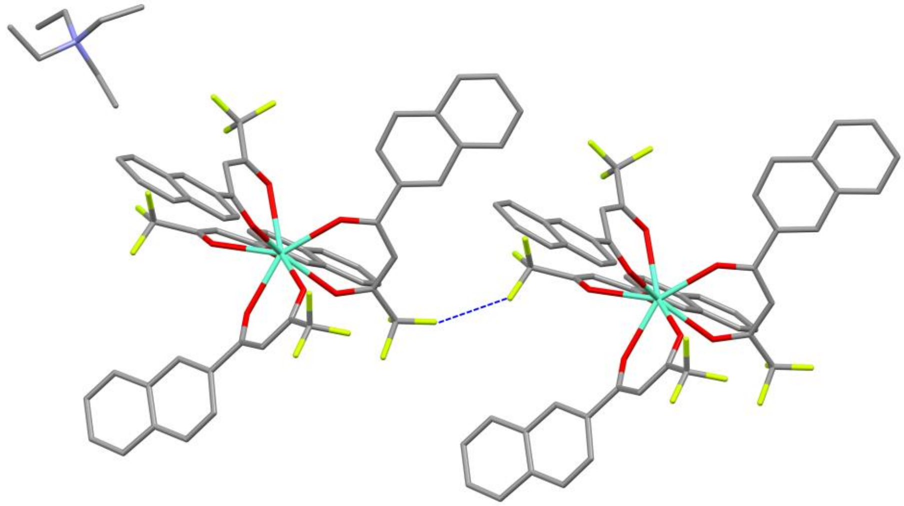

Figure S6. Intermolecular F···F contacts (blue dashed lines) in $(Et_4N)[Eu(nta)_4]$ crystal. The F···F contact has distance of 2.741 Å and angle of 150.70°.

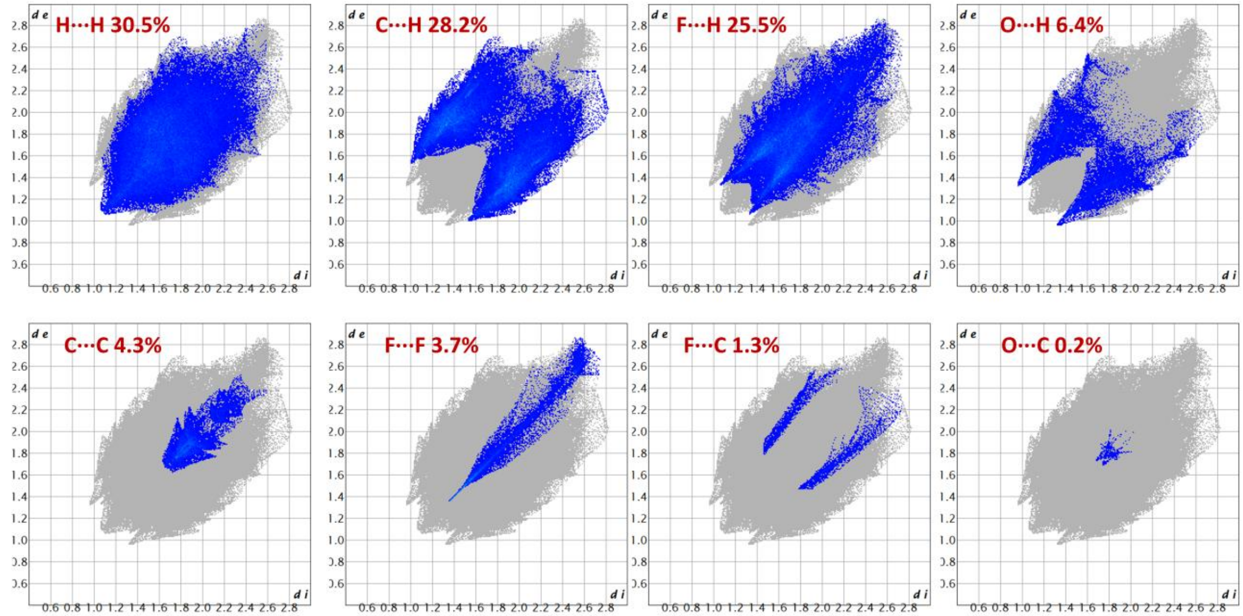

Figure S7. 2D fingerprint plots of interatomic interactions in $(Et_4N)[Eu(nta)_4]$ crystal, showing the percentages of contacts contributed to the total Hirshfeld surface area of the molecules.

Supplementary note S4 – Powder X-ray diffraction of the complexes

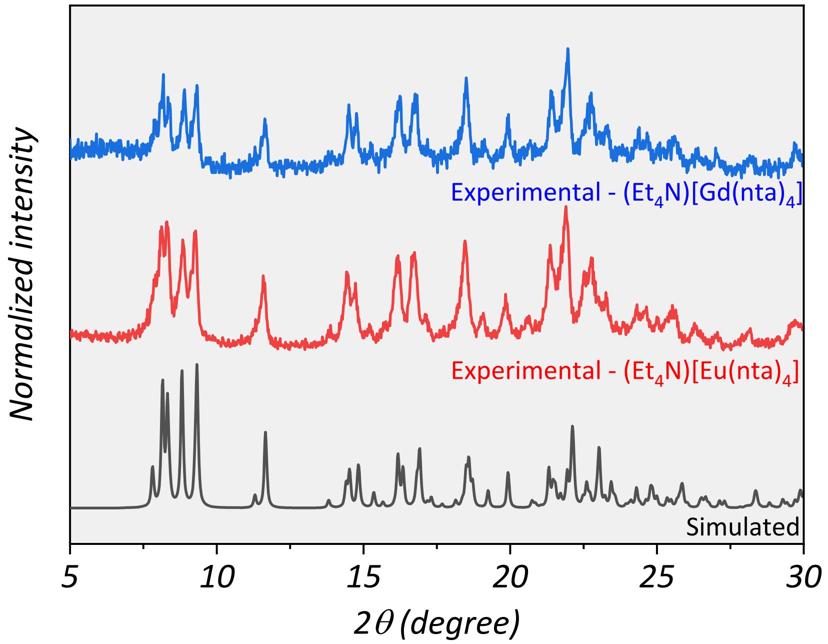

Figure S8. Experimental PXRD pattern of $(Et_4N)[Eu(nta)_4]$ and $(Et_4N)[Gd(nta)_4]$ compared to the PXRD pattern simulated from SC-XRD analysis of the $(Et_4N)[Eu(nta)_4]$ crystal. The experimental PXRD were collected for the ground crystals at 298 K.

Supplementary note S5 – Further spectroscopic data of the $(Et_4N)[Ln(nta)_4]$ complexes as crystals

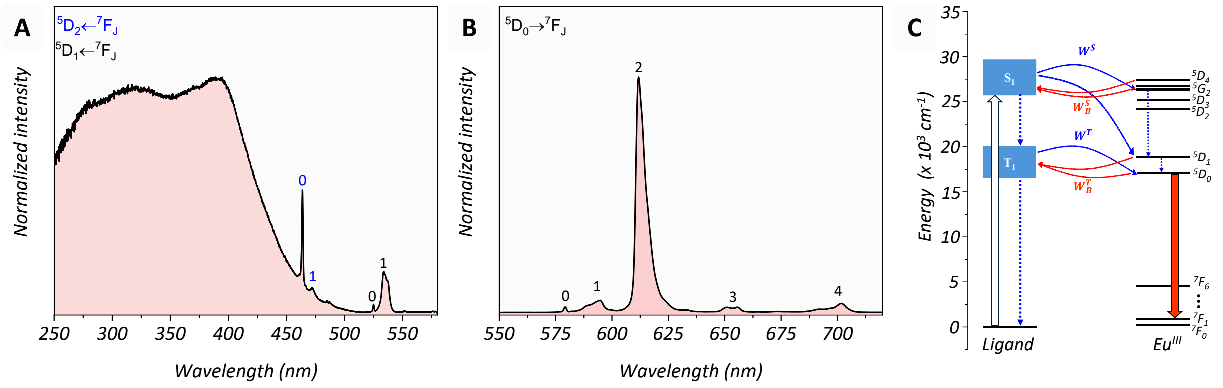

Figure S9. A) Excitation (λ_{em} = 613 nm) and B) emission (λ_{exc} = 390 nm) spectra of the $(Et_4N)[Eu(nta)_4]$ crystal measured at 300 K. C) Partial energy level diagram showing the primary energy transfer pathways for the $(Et_4N)[Eu(nta)_4]$ complex. S_1 and T_1 are the ligand singlet and triplet excited states, respectively. W^S and W^T are the singlet and triplet intramolecular energy transfer (IET) rates, respectively, and W_B^S and W_B^T are the intramolecular back energy transfer (IBET) rates.

Singlet ad triplet state energies determined from the $(Et_4N)[Gd(nta)_4]$ complex

The triplet state (T_1) energy position was estimated by using the $(Et_4N)[Gd(nta)_4]$ specie as a spectroscopic reference. In Gd^{III} complexes, the $^6P_{7/2}$ excited level presents high energy (at about 32,000 cm^{-1}); as consequence, there is no possibility of ligand-to-Gd^{III} energy transfer, and the observed phosphorescence arises predominantly from the ligand triplet state.[3,4] The Gd^{III} emission spectrum is dominated by a weak and broad band characteristic of β-diketonate-centered emission (Figure S10). The zero-phonon transition was determined as 19,193±18 cm^{-1} and assumed as the triplet excited state (T_1). This value confirms that the T_1 state is adequate for sensitizing the Eu^{III} luminescence[5], as further discussed by luminescence spectroscopy. The S_1 state energy was determined as 26,095 cm^{-1} from the maximum of the band at lower energy from the diffuse reflectance spectrum of $(Et_4N)[Gd(nta)_4]$ complex (Figure S11).

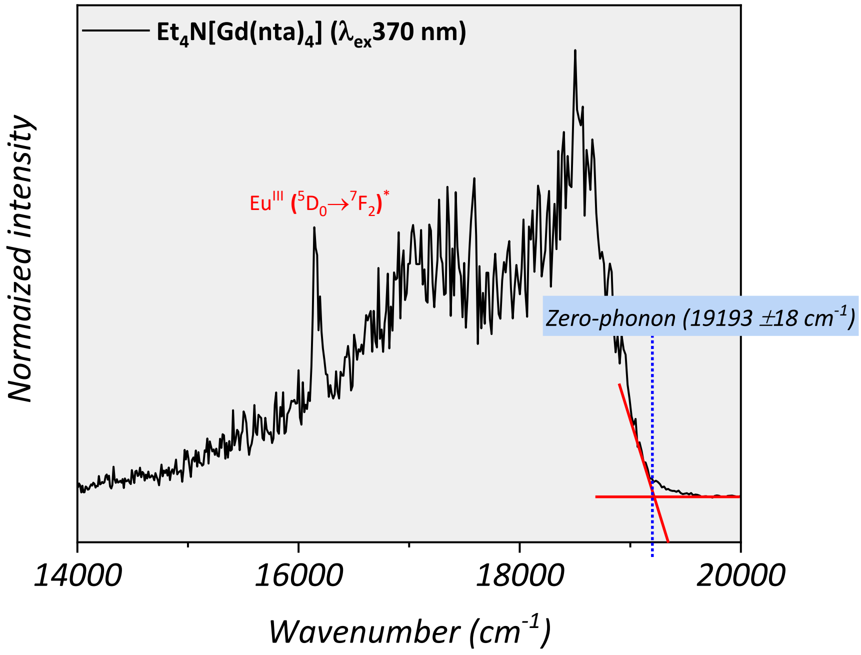

Figure S10. Emission spectrum of $(Et_4N)[Gd(nta)_4]$ measured at 77 K. The spectrum was used to determine the triplet state energy (T_1) of the complex by using the zero-phonon energy method. *Contamination with Eu^{III} ion (probably from the Gd_2O_3 oxide used in the synthesis) evidenced by $^5D_0 \rightarrow ^7F_2$ transition.

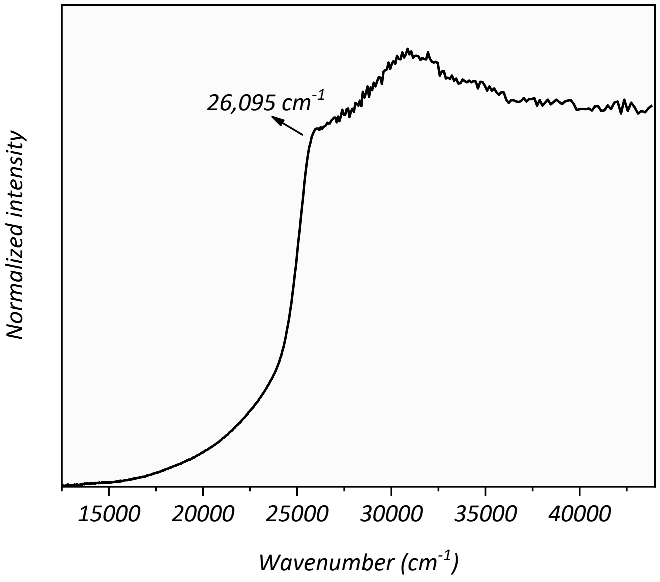

Figure S11. Diffuse reflectance spectrum of $(Et_4N)[Gd(nta)_4]$ obtained at 300 K. The maximum of the lower energetic band of the DRS spectrum was assumed as the S_1 state energy of the complex.

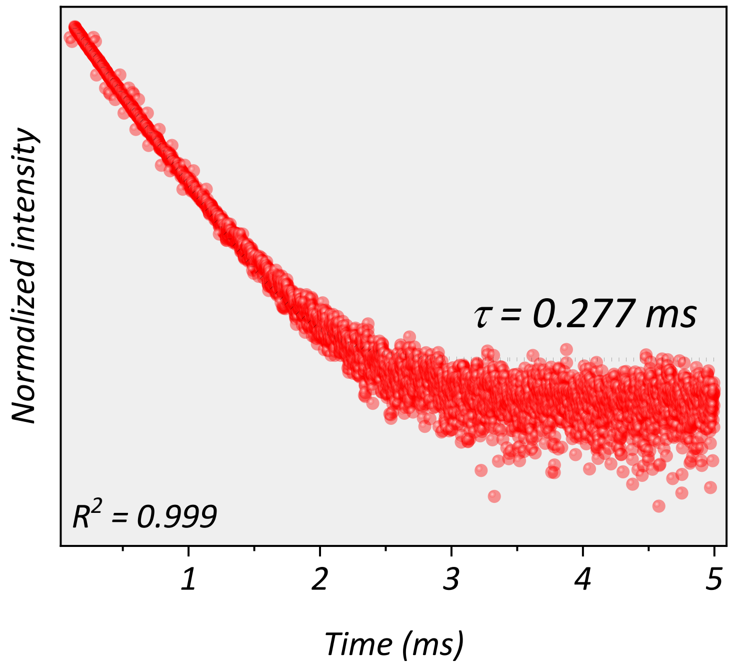

Figure S12. Emission decay curves used to extract the $^5D_0$ level lifetime of $(Et_4N)[Eu(nta)_4]$ at 300 K (black dashed line represents the data best fitting to a monoexponential decay function, $R^2 > 0.99$).

Supplementary note S6 – Calculations of the experimental photophysical properties of Eu^{III}

The radiative decay rate (A_{rad}) of the Eu^{III} $^5D_0$ level was calculated from eqn S2 and S3, where A is the Einstein coefficient of spontaneous emission. In eqn S2, the term $I_{0\text{-}\lambda}$ is the area under the curve related to the $^5D_0 \rightarrow {}^7F_\lambda$ transition, $h\nu_{0-\lambda}$ is the energetic barycenter of the 0–λ transition, and $A_{01} = 14.65\ n^3$ in s^{-1}, where n is the refractive index used as 1.500 for the powder complex and 1.409 for the PMMA films.[6] The non-radiative decay rate (A_{nrad}) of the Eu^{III} $^5D_0$ level was calculated from eqn S4, where τ is the $^5D_0$ level lifetime. Finally, the intrinsic Eu^{III} emission quantum yield (Q_{Eu}^{Eu}) was determined from eqn S5. The experimental intensity parameters Ω_λ (λ = 2 and 4) were calculated by using eqn S6.

$$A_{0-\lambda} = A_{01} \frac{I_{0-\lambda}}{I_{0-1}} \frac{h\nu_{0-1}}{h\nu_{0-\lambda}} \tag{S2}$$

$$A_{rad} = \sum_J A_{0-J} \tag{S3}$$

$$A_{total} = \frac{1}{\langle \tau \rangle} = A_{rad} + A_{nrad} \tag{S4}$$

$$Q_{Eu}^{Eu} = \frac{A_{rad}}{A_{rad} + A_{nrad}} \tag{S5}$$

$$\Omega_\lambda = \frac{3hc^3 A_{0\lambda}}{8\pi e^2 \omega^3 \chi \left| \langle {}^7F_J \| U^{(\lambda)} \| {}^5D_0 \rangle \right|^2} \tag{S6}$$

Supplementary note S7 – Computational calculations of the luminescence dynamics of $(Et_4N)[Ln(nta)_4]$ complexes as crystals

Theoretical intensity parameters. The theoretical data obtained from JOYSPECTRA[7] website, employed structural parameters obtained from SC-XRD without any DFT optimization, intensity parameters (Ω_2 and Ω_2) derived from the emission spectrum, the $^5D_0$ emission lifetime of the $(Et_4N)[Eu(nta)_4]$ complex measured at 300 K, and the singlet and triplet state energies determined from the Gd^{III} analog.

The theoretical intensity parameters Ω_λ (λ = 2, 4, and 6) were determined following the model reported in references 8,9,10,11. The values of Ω_λ are influenced by the chemical environment surrounding the lanthanide(III) ion, as described by:

$$\Omega_\lambda = (2\lambda + 1)\sum_{t,p} \frac{|B_{\lambda tp}|^2}{(2t+1)} \tag{S7}$$

where t (= 1, 3, 5, and 7) and p ($-t \le p \le +t$) represent the rank and component, respectively, that define the spherical harmonics in the expression of $B_{\lambda tp}$. In systems lacking an inversion center, the two major contributions to $B_{\lambda tp}$ arise from the forced electric dipole (FED) and dynamic coupling (DC) mechanisms, as described below:

$$B_{\lambda tp} = B_{\lambda tp}^{FED} + B_{\lambda tp}^{DC} \tag{S8}$$

$$B_{\lambda tp}^{FED} = \frac{2}{\Delta E}\langle r^{t+1}\rangle \Theta(t,\lambda)\gamma_p^t \tag{S9}$$

$$B_{\lambda tp}^{DC} = -\left[\frac{(\lambda+1)(2\lambda+3\_}{(2\lambda+1)}\right]^{\frac{1}{2}} \langle r^\lambda\rangle \langle f||C^{(\lambda)}||f\rangle \Gamma_p^t \delta_{t,\lambda+1} \tag{S10}$$

The $\langle r^k\rangle = \langle 4f|r^k|4f\rangle$ terms correspond to the 4f radial integrals[12,13,14], and ΔE denotes the energy gap between the barycenter of the excited $4f^{n-1}5d^1$ and ground $4f^n$ configurations, as defined by the average energy denominator method[15]. The Θ(t, λ) are numerical coefficients obtained according to Reference [16]:

$$\Theta(t,\lambda) = \langle f||C^{(1)}||g\rangle\langle g||C^{(t)}||f\rangle \begin{Bmatrix} f & t & g \\ 1 & f & \lambda \end{Bmatrix} + (1 - 2\delta_t)\langle f||C^{(1)}||d\rangle\langle gd||C^{(t)}||f\rangle \begin{Bmatrix} f & t & g \\ 1 & f & \lambda \end{Bmatrix} \tag{S11}$$

The Θ(t, λ) values include contributions from opposite-parity orbital interactions, namely $f \rightarrow g$ (first term) and $f \rightarrow d$ (second term). The parameter δ_t represents the fractional contribution arising from core excitations of the type $4f \rightarrow nd$ (n = 3 and 4).

$$\delta_t = \frac{1}{\langle 4f|r^{t+1}|4f\rangle}\sum_{n=3,4}\langle 4f|r|nd\rangle\langle 4f|r^t|nd\rangle \tag{S12}$$

Equation S12 establishes a relation between the radial integrals, and the values of δ_t cannot exceed 1, which becomes evident when applying the closure relation for a complete basis set, $\sum_n |nd\rangle\langle nd| = 1$. Hartree–Fock calculations[13,17] yielded δ_1 = 0.539, δ_3 = 0.223, δ_5 = 0.082, and $\delta_7 \approx 0$. Consequently, the Θ(t, λ) values are: Θ(1,2) = −0.17; Θ(3,2) = 0.34; Θ(3,4) = 0.18; Θ(5,4) = −0.24; Θ(5,6) = −0.24; and Θ(7,6) = 0.24.

The quantities γ_p^t (ligand-field terms) and Γ_p^t (polarizability-dependent terms α_{OP} and α') are determined by the structural characteristics and the chemical environment surrounding the lanthanide ion. These expressions are derived from the Simple Overlap Model for the ligand-field contribution of the FED mechanism (Equation S13)[18,19], and from the Bond Overlap Model for the polarizability-dependent contribution of the DC mechanism (BOM, Equation S14)[9].

$$\gamma_p^t = e^2 \left(\frac{4\pi}{2t+1}\right)^{\frac{1}{2}} \sum_j \rho_j \left(2\beta_j\right)^{t+1} \frac{g_j}{R_j^{t+1}} Y_p^{t^*}\left(\theta_j, \phi_j\right) \quad \text{(S13)}$$

$$\Gamma_p^t = \left(\frac{4\pi}{2t+1}\right)^{\frac{1}{2}} \sum_j \frac{\left[\left(2\beta_j\right)^{t+1} \alpha_{OP,j} + \alpha'_j\right]}{R_j^{t+1}} Y_p^{t^*}\left(\theta_j, \phi_j\right) \quad \text{(S14)}$$

where e is the elementary charge, the ρ terms correspond to the Ln–ligand overlap integrals, and $\beta_j = 1/(1 \pm \rho)$ depends on ρ[20]. The complex conjugate of the spherical harmonics is denoted by $Y_p^{t^*}\left(\theta_j, \phi_j\right)$. The summation over j includes all ligands in the first coordination sphere.

The DC mechanism was originally formulated using the isotropic dipole polarizability approximation for the coordinating atoms[21,22,23,24]. The BOM represents a refinement of the original DC model by separating the contributions of overlap polarizabilities (α_{OP}) and the effective polarizabilities of the ligating atoms (α′). It is important to note that shielding factors do not appear in the BOM, since this effect is already incorporated into the definition of α_{OP}.

Application of the BOM requires estimating α_{OP} for the Ln–ligand chemical bonds. For this purpose, α_{OP} can be expressed as a power series expansion in the total squared overlap integrals, ρ, between the valence orbitals of Ln^{III} and the donor atoms. The expression for the overlap polarizability (α_{OP}) is given in Reference. [25, 26]:

$$\alpha_{OP} = \frac{e^2 \rho^2 R^2}{2\Delta\varepsilon} \quad \text{(S15)}$$

Intramolecular energy transfer rates. The intramolecular energy-transfer (IET) rates from the ligands to the Ln^{III} ion were determined by considering contributions from the dipole–dipole ($W_{d\text{-}d}$), dipole–multipole ($W_{d\text{-}m}$), and exchange (W_{ex}) mechanisms [8,27,28,29,30].

$$W_{d-d} = \frac{S_L(1-\sigma_1)^2}{(2J+1)G} \frac{4\pi}{\hbar} \frac{e^2}{R_L^6} \sum_\lambda \Omega_\lambda^{FED} \left\langle \psi' J' \middle\| U^{(\lambda)} \middle\| \psi J \right\rangle^2 F \quad \text{(S16)}$$

$$W_{d-m} = \frac{S_L}{(2J+1)G}\frac{2\pi e^2}{\hbar}\sum_\lambda(\lambda+1)\frac{\langle r^\lambda\rangle^2}{\left(R_L^{\lambda+2}\right)^2}\langle f||C^{(\lambda)}||f\rangle^2(1-\sigma_\lambda)^2 \times \langle\psi'J'||U^{(\lambda)}||\psi J\rangle^2 F \quad \text{(S17)}$$

$$W_{ex} = \frac{(1-\sigma_0)^2}{(2J+1)G}\frac{8\pi}{\hbar}\frac{e^2}{R_L^4}\langle\psi'J'||S||\psi J\rangle^2\sum_m\left|\langle\phi|\Sigma_j\mu_z(j)S_m(j)|\phi^*\rangle\right|^2 F \quad \text{(S18)}$$

The donor-acceptor distance, R_L, corresponds to the separation between the ligand and lanthanide energy levels. The Ω_λ^{FED} parameters represent the intensity factors associated exclusively with the forced electric-dipole mechanism (computed using only Eq. S11 for Ω_λ) and were obtained using the superposition model (SOM) for the ligand field[18,19]. The squared reduced matrix elements $\langle\psi'J'\|U(\lambda)\|\psi J\rangle^2$ quantities that depend solely on the Ln^{III} ion were taken from Carnall et al.[31]. S_L denotes the dipole strength of the ligand transition involved in the IET process (approximately ~10^{-36} and ~10^{-40} $(esu)^2\cdot cm^2$ for S_1 and T_1, respectively[8]). The $\langle r^\lambda\rangle$ values correspond to the 4f radial integrals; G is the ligand-state degeneracy (G = 1 for S_1 and 3 for T_1); $\langle f\|C(\lambda)\|f\rangle$ (with f = 3) represents the reduced matrix element of Racah tensor operators; and the shielding factors (1 − $\sigma\lambda$) account for the overlap between the valence orbitals of the Ln–X pair (where X is the coordinating atom in the first coordination sphere) [20,30].

In Equation S18, S_m is the ligand spin operator and μz is the z-component of the dipole operator; the matrix element of this coupled operator is on the order of ~10^{-36} $(esu)^2\cdot cm^2$.[8,32] The reduced matrix elements $\langle\psi'J'\|S\|\psi J\rangle$ of the lanthanide spin operator were calculated using free-ion wavefunctions under the intermediate-coupling approximation[33].

The factor F in the preceding equations corresponds to the spectral overlap term, which accounts for the energy mismatch between donor and acceptor states[8,28]. For ligand-to-Ln^{III} intramolecular energy transfer, F can be estimated as[8]:

$$F = \frac{1}{\hbar\lambda_L}\sqrt{\frac{\ln(2)}{\pi}}e^{-\left(\frac{\Delta}{\hbar\lambda_L}\right)^2\ln(2)} \quad \text{(S19)}$$

where Δ represents the energy gap between the donor state and the lanthanide acceptor level, defined as $\Delta = E_{lig} - E_{Ln}$. The term γL corresponds to the full width at half maximum (FWHM) of the ligand state. This expression applies under the condition that γL is significantly larger than the FWHM of the Ln^{III} ion energy levels.

The forward energy-transfer rates (W), in which the lanthanide ion acts as the acceptor, are obtained by summing the contributions from Equation. S16, S17, and S18 along the same transfer pathway:

$$W = W_{d-d} + W_{d-m} + W_{ex} \quad \text{(S20)}$$

The rate W must be multiplied by the barrier factor $\exp(-|\Delta|/k_{BT})$ only when Δ is negative, where k_B is the Boltzmann constant and T is the temperature. The same equations and

considerations are applied to determine the backward IET rates, W_b, which describe energy transfer from the Ln^{III} ion back to the ligand states.

Data extracted from JOYspectra calculations

To gain insight into the energy transfer processes in the $(Et_4N)[Eu(nta)_4]$ complex, the *JOYSpectra platform*[34] was used to calculate the energy transfer rates. The calculations enable the determination of the charge factors (g) and polarizabilities (α) data of the oxygen atoms directly coordinated to Eu^{III}, along with the non-radiative rates of nta-to-Eu^{III} intramolecular energy-transfer (IET). The polarizability (α) of each coordinating atom includes contributions from both the effective (α′) and overlap (α_{OP}) components, such that $\alpha = \alpha' + \alpha_{OP}$ (Table S7). Values of g and α′ were obtained for the oxygen atoms of the bidentate nta^- ligands, classified into two groups: those adjacent to the CF_3 group and those near the naphthalene (naft) moiety. In the calculations, the *JOYSpectra platform* iteratively adjusted the g and α′ values to minimize the difference between the theoretical ($\Omega_\lambda^{Theor.}$) and experimental ($\Omega_\lambda^{Exp.}$) intensity parameters (λ = 2, 4, 6). The small discrepancy between the theoretical and experimental values (Table S6) confirms the high quality of the obtained fit.

The polarizabilities of Eu-O bonds in the Eu^{III} first coordination sphere were evaluated from the α′ and α_{OP} values of the oxygen atoms of the nta^- ligands. For oxygen atoms adjacent to the naft group, shorter bond distances (as evidenced by SC-XRD data) and lower g/α_{OP} values were obtained compared to those near the CF_3 moiety side (Table S7). This trend arises from the electron-withdrawing character of the CF_3 group, which reduces local polarizability, whereas the electron-donating naphthalene group enhances it in the Eu^{III} coordination environment.

The ligand-to-Eu^{III} IET rates were determined from the forced electric dipole contributions (Ω_λ, λ = 2, 4, and 6) calculated using the *JOYSpectra platform*.[35,36,37,38] The dominant IET rates were estimated considering dipole-multipole (W_{dm}) and exchange (W_{ex}) mechanisms. It is noteworthy that the dipole-dipole (W_{dd}) and dipole-multipole (W_{dm}) contributions were totally negligible compared to the exchange contributions. All these energy transfer channels are summarized in Figure S11C.

Table S6. Experimental and theoretical intensity parameters (Ω_λ, λ = 2,4 and 6) and their contributions of Forced Electric Dipole (FED) and Dynamic coupling (DC) (in 10^{-20} cm^2).

λ	$\Omega^{Exp.}$	$\Omega^{Teo.}$	$\Omega^{FED.}$	$\Omega^{DC.}$
**Ω_2**	63.94	63.97	0.001043	63.71
**Ω_4**	5.9	8.97	0.012102	8.33
**Ω_6**	-	0.99	0.022642	0.83

Table S7. Bond distance (R, Å), polarizabilities (α), electron density of the integral (ρ), and charge factors (g) for $(Et_4N)[Eu(nta)_4]$ considering the oxygen atoms in the coordination sphere of Eu^{III}. In the first column, the notations within the parentheses are the equivalent labels assigned in the SC-XRD measurement.

Atom#	Atom	R(Å)	α_{OP}	ρ	g factor
**2 (O1b)**	O_{-naft}	2.354862	0.04032	0.06877	0.136
**3 (O2d)**	O_{-naft}	2.346173	0.04095	0.06965	0.136
**5 (O1b)**	O_{-CF3}	2.422510	0.03566	0.0623	0.186
**6 (O1a)**	O_{-CF3}	2.452339	0.03373	0.05963	0.186
**8 (O1c)**	O_{-CF3}	2.410550	0.03645	0.0634	0.186
**11 (O1d)**	O_{-CF3}	2.432388	0.03501	0.06141	0.186
**12 (O2b)**	O_{-naft}	2.337992	0.04155	0.07048	0.136
**17 (O2c)**	O_{-naft}	2.341801	0.04127	0.07009	0.136

Table S8. Direct IET rates (W^S and W^T) and back IET rates (W_B^S and W_B^T) in units of s^{-1} for $(Et_4N)[Eu(nta)_4]$ complex as crystals, considering the contributions of dipole–multipole (W_{dm}) and exchange (W_{ex}) mechanisms.

**Intramolecular Energy Transfer (IET)**			
**Energy transfer from S_1 State**	W (%)	W_{total} (s^{-1})	W_{ex} (s^{-1})
**$S_1 \rightarrow (^7F_0 \rightarrow {}^5D_1)$**	72.4	7.59×10^9	7.59×10^9
**$S_1 \rightarrow (^7F_1 \rightarrow {}^5G_2)$**	19.6	2.06×10^9	2.06×10^9
**Back energy transfer from S_1 state**			
**$S_1 \leftarrow (^7F_1 \rightarrow {}^5G_2)$**	98.7	5.17×10^9	5.17×10^9
**Energy transfer from T_1 State**			
**$T_1 \rightarrow (^7F_1 \rightarrow {}^5D_0)$**	99.9	6.22×10^9	6.22×10^9
**Back energy transfer from T_1 state**			
**$T_1 \leftarrow (^7F_1 \rightarrow {}^5D_1)$**	33.0	7.78×10^9	7.78×10^9
**$T_1 \leftarrow (^7F_1 \rightarrow {}^5D_0)$**	64.4	1.52×10^{10}	1.52×10^{10}

Supplementary note S8 – Characterization of the PMMA:Eu^{III} films

Table S9. Composition of the films.

Name	PMMA		$(Et_4N)[Eu(nta)_4]$		
	**Wt. (%)**	**Mass (g)**	**Wt. (%)**	**Mass (g)**	**Thickness (mm)**
**PMMA**	100%	0.3000	-	-	0.23
**PMMA:1%Eu^{III}**	99%	0.2970	1%	0.0030	0.24
**PMMA:2%Eu^{III}**	98%	0.2940	2%	0.0060	0.23
**PMMA:3%Eu^{III}**	97%	0.2910	3%	0.0090	0.22
**PMMA:4%Eu^{III}**	96%	0.2880	4%	0.0120	0.24
**PMMA:5%Eu^{III}**	95%	0.2850	5%	0.0150	0.24

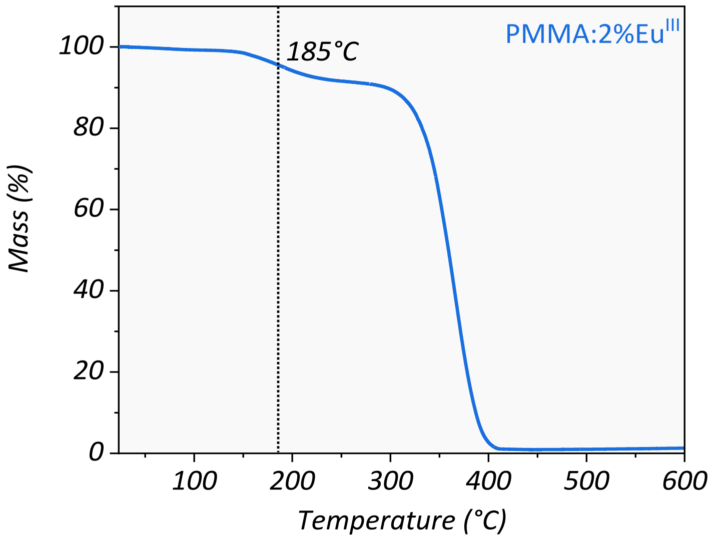

Figure S13. Thermogravimetric analysis of PMMA:2%Eu^{III}.

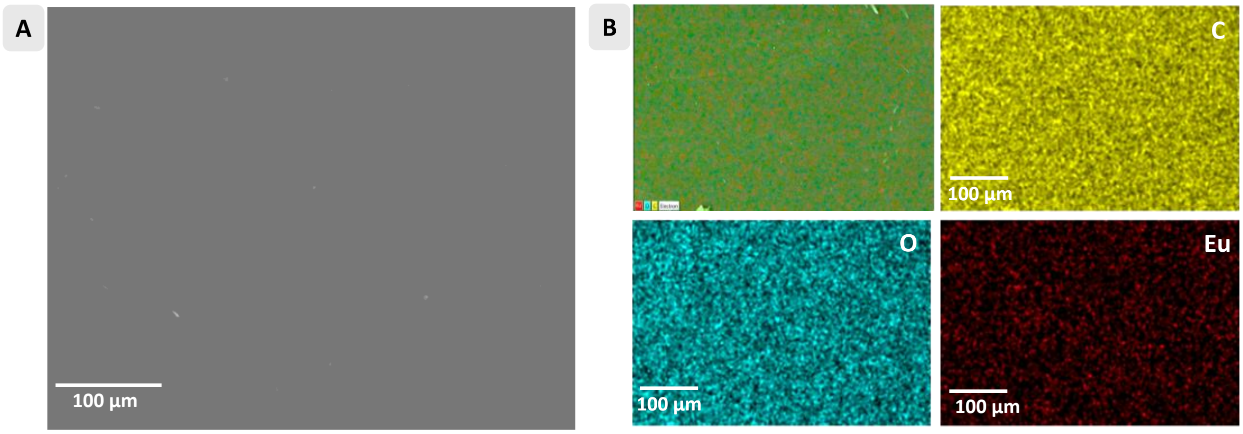

Figure S14. A) SEM image of the PMMA:2%Eu film. (B) Chemical mapping of the PMMA:5%Eu film. The first image in (B) is the merge among the C, O, and Eu mapping images.

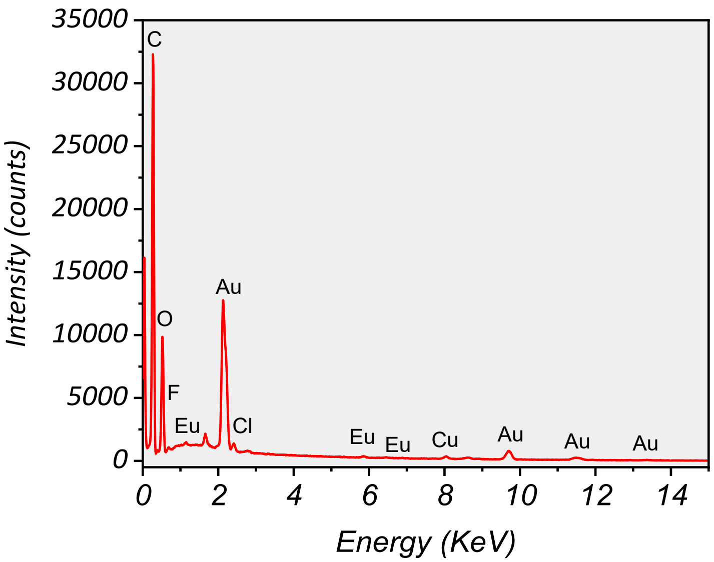

Figure S15. Energy dispersive spectroscopy (EDS) of the PMMA:5%Eu^{III} film highlighting the assignments of the detected chemical elements. To undertake the EDS and chemical mapping, Au was used as coating.

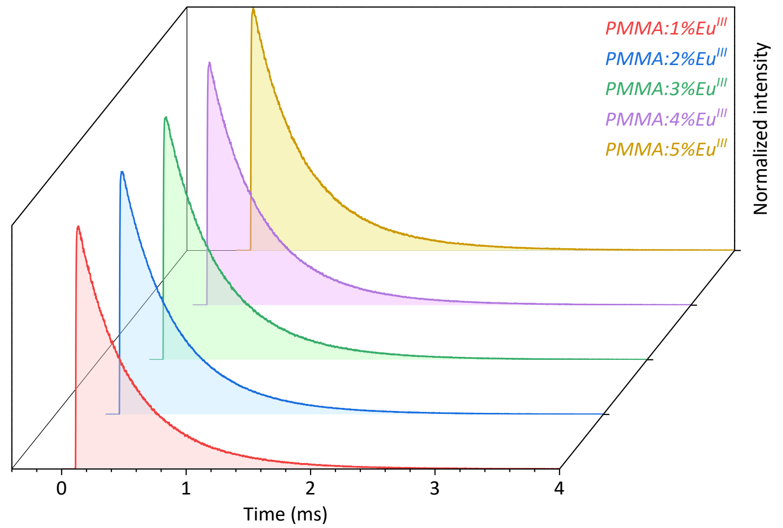

Figure S16. Emission decay curves (300 K) used to extract the $^5D_0$ level lifetime of PMMA films doped with $(Et_4N)[Eu(nta)_4]$ complex at different weight amount. λ_{ex} = 390 nm and λ_{em} = 613 nm.

Supplementary note S9 – Stability tests of PMMA:2%Eu^{III} film

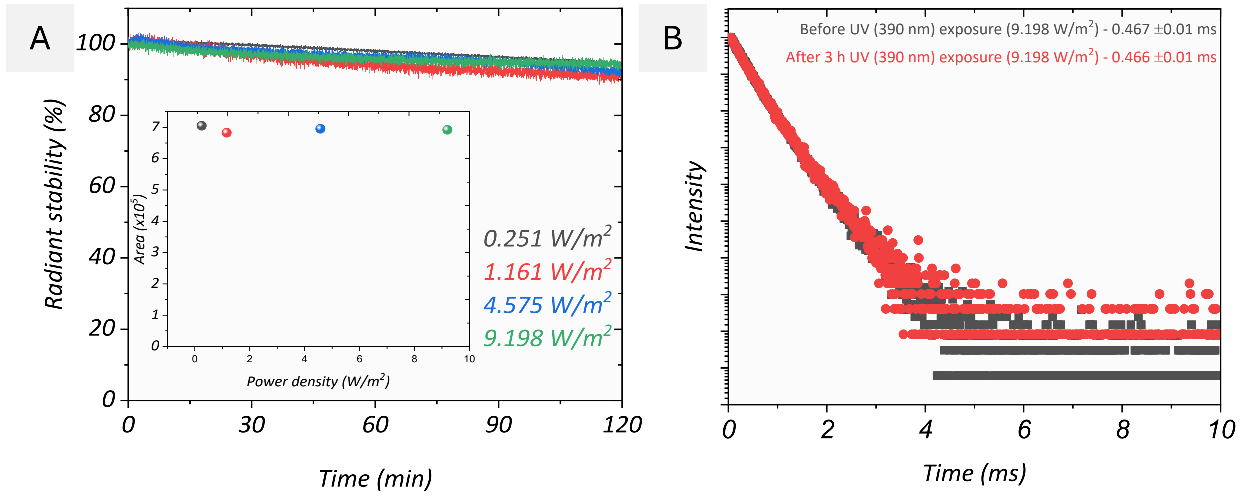

Figure S17. A) Photostability test of the 2% film undertaken by monitoring the Eu^{III} emission at 613 nm upon 390 nm excitation for 2 hours under different power density irradiation. B) Emission decay curves (300 K) used to extract the $^5D_0$ level lifetime of PMMA:2%Eu^{III} film before and after 3 h of UV (390 nm) exposure.

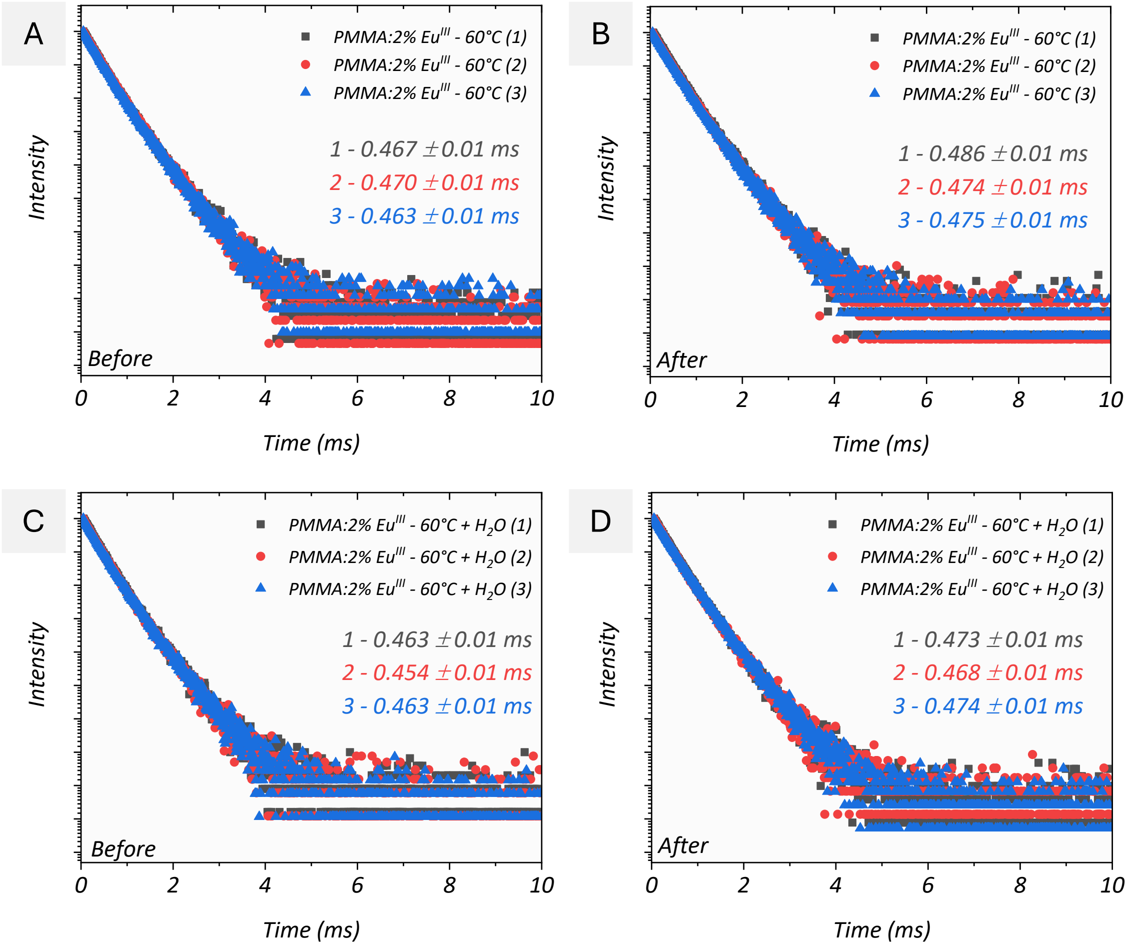

Figure S18. Emission decay curves measured at 300 K at three different points of the PMMA:2%Eu^{III} film used to determine the $^5D_0$ excited-level lifetime: A) before and B) after exposure at 60 °C for 24 h; and C) before and D) after exposure to 60 °C while immersed in water for 24 h.

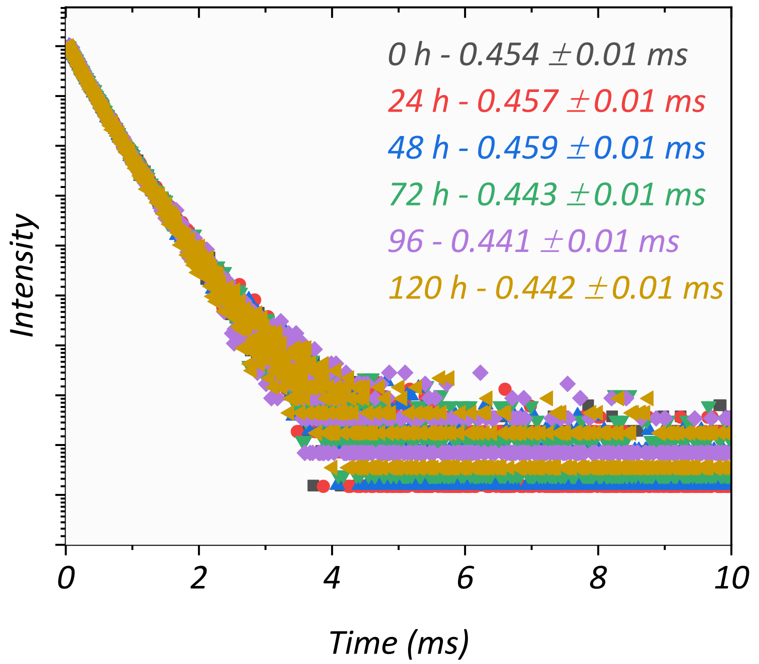

Figure S19. Emission decay curves (300 K) used to extract the $^5D_0$ level lifetime of PMMA:2%Eu^{III} film after daily exposure to solar irradiation over five consecutive days.

Supplementary note S10 – LSC tests

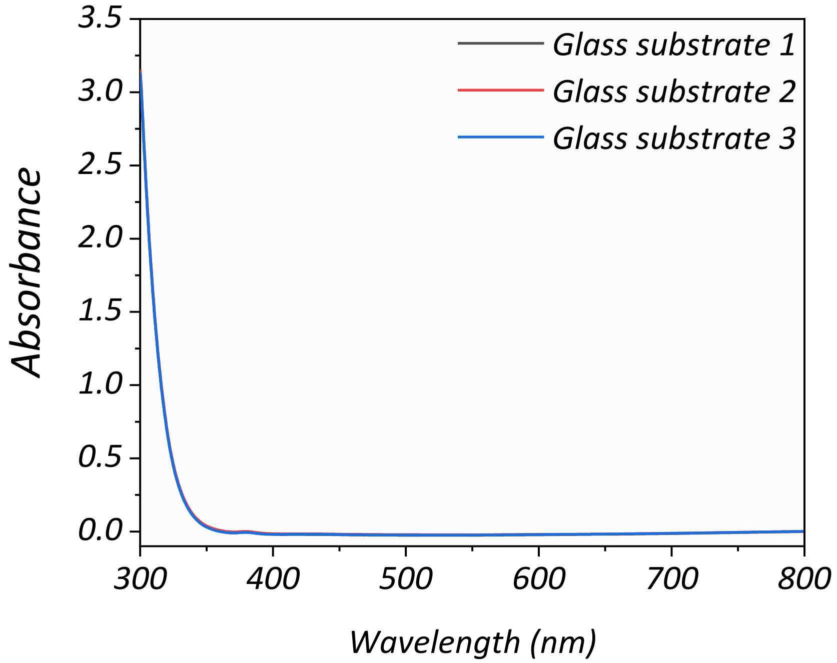

Figure S20. UV-vis spectra of all glass substrate used in the current measurements.

Table S10. Solar irradiance power and current data acquired in the LSC tests (between 8:00 AM and 1:00 PM).

		Current (mA)			
	**Solar Irradiance Power (W/m²)**	Bare Si PV	c-Si PV + Glass Substrate	c-Si PV + Glass substrate + PMMA	c-Si PV + Glass substrate + PMMA:2%Eu^{III}
8:00 AM	7.537	0.292	0.113	0.118	0.137
	7.565	0.301	0.114	0.124	0.134
	7.657	0.305	0.114	0.121	0.139
Average	7.565	0.299	0.114	0.121	0.137
SD	0.063	0.007	0.001	0.003	0.003
9:00 AM	8.107	0.263	0.115	0.114	0.142
	9.238	0.308	0.158	0.164	0.168
	10.120	0.532	0.250	0.253	0.281
Average	9.238	0.368	0.174	0.177	0.197
SD	1.009	0.144	0.069	0.070	0.0738
10:00AM	12.090	0.677	0.283	0.371	0.426
	11.647	0.558	0.228	0.270	0.370
	10.300	0.480	0.234	0.205	0.290
Average	11.647	0.571	0.248	0.282	0.362
SD	0.932	0.099	0.0301	0.084	0.068
11:00 AM	38.800	2.410	0.64	0.774	0.822
	39.891	2.450	0.68	0.87	1.108
	40.230	2.460	0.869	1.058	1.160
Average	39.891	2.440	0.729667	0.900667	1.03
SD	0.747	0.026458	0.122312	0.144462	0.182
12:00 PM	23.900	0.524	0.256	0.361	0.377
	26.422	0.681	0.290	0.347	0.401
	22.910	0.647	0.270	0.363	0.460
Average	23.900	0.617333	0.272	0.357	0.412667
SD	1.810	0.082597	0.017088	0.008718	0.042712
1:00 PM	14.936	0.468	0.220	0.236	0.286
	11.176	0.373	0.178	0.220	0.272
	10.265	0.358	0.174	0.247	0.298
Average	11.176	0.399	0.191	0.234	0.285
SD	2.476	0.059	0.025	0.013	0.013

Table S11. Solar irradiance power and current data acquired in the LSC tests (between 2:00 PM and 4:00 PM).

		Current (mA)			
	**Solar Irradiance Power (W/m^2)**	Bare Si PV	c-Si PV + Glass Substrate	c-Si PV + Glass substrate + PMMA	c-Si PV + Glass substrate + PMMA:2%Eu^{III}
2:00 PM	8.577	0.845	0.262	0.427	0.548
	9.757	0.984	0.292	0.455	0.638
	10.771	1.297	0.341	0.542	0.709
Average	9.757	1.042	0.298	0.475	0.632
SD	1.098	0.231	0.040	0.060	0.081
3:00 PM	10.971	0.874	0.269	0.343	0.422
	9.731	0.958	0.267	0.347	0.405
	9.207	0.946	0.277	0.281	0.467
Average	9.731	0.926	0.271	0.324	0.431
SD	0.906	0.045	0.005	0.037	0.032
4:00 PM	6.225	0.579	0.207	0.221	0.270
	5.886	0.491	0.184	0.223	0.236
	5.700	0.518	0.195	0.205	0.230
Average	5.886	0.529	0.195	0.216	0.245
SD	0.266	0.045	0.011	0.009	0.021

Table S12. Comparison of Photoluminescence Quantum Yield (PLQY) and Optical Conversion Efficiency (η_{opt}) for Selected Luminescent Solar Concentrator (LSC) Prototypes Reported in Literature.

**Architecture**	**PLQY (%)**	**Device Dimensions**	**η_{opt} (%)**	**Ref.**
F4Eu/F4Tb, Thin Film, Glass	-	-	1.2/1.7	39
LR305/Urethane matrix (LT), Thin Film, Glass	-	-	2.48	40
CuInSexS2-x/ZnS QD, Doped, poly(lauryl methacrylate)	40	12 × 12 × 0.3	3.27	41
**PMMA:2%Eu**	**49**	**5 x 5 x 0.23**	**3.4**	**This work**
DCJTB, Pt(TPBP), Thin Film, Glass	-	2.5 x 2.5 x 0.2	4.7	42
PMMA:80% $[Eu(tta)_4]$tpp	62.5	3 x 3 x 0.4	5.4	43
PbS/CdS QD, Doped, Acrylate	≈40–50	5 x 1.5 x 0.3	6.1	44
CdSe/CdS QD, Doped, PMMA	45	21.5 x 1.3 x 0.5	10.2	45
Dye: 4CzIPN	-	7.5 x 7.5 x 0.3	10.4	46
Eu^{III} bridged silsesquioxane, Thin Film, Glass	23	-	12.3	47
TPE/PMMA, Thin Film, Glass	49.5	1 x 1 x 0.1	13.2	48
LR305, Doped, Di-ureasil	97	4.4 × 4.4 × 0.3	14.5	49
CQDs/PVP	-	5 x 5 x 0.3	16.32	50

F4: silsesquioxane; LR305: Lumogen F Red 305; DCJTB: 1,1,7,7-tetramethyljulolidyl-9-enyl)-4H-pyran; Pt(TPBP): platinum tetraphenyltetraben-zoporphyrin; tta: 2-Thenoyltrifluoroacetone; tpp: tetraphenylphosphonium; 4CzIPN: 1,2,3,5-tetrakis(carbazol-9-yl)-4,6-dicyanobenzene; TPE: tetraphenylethene.

Supplementary note S11 – Lifetime Thermometry of PMMA:2%Eu^{III} film

The relative thermal sensitivity of the complex was calculated from eqn S21, where *Δ* is the thermometric parameter ($^5D_0$ level lifetime) and *T* is the temperature. The temperature uncertainty (δT) is calculated by eqn S22.[51] δΔ/Δ is the uncertainty in the lifetime determination and it was considered as 0.01 ms over the entire temperature range.

$$S_r = \frac{1}{\Delta}\left|\frac{d\Delta}{dT}\right| \quad \text{(S21)}$$

$$\delta T = \frac{1}{S_r}\frac{\delta\Delta}{\Delta} \quad \text{(S22)}$$

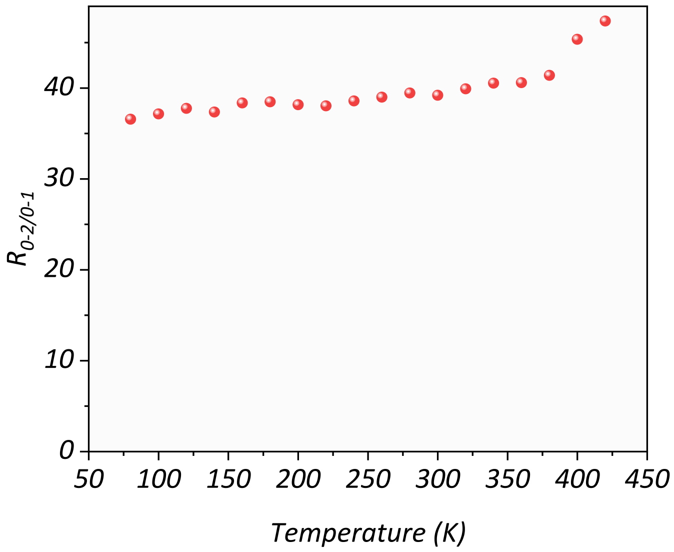

Figure S21. Temperature dependency of the intensity ratio between the $^5D_0 \rightarrow ^7F_2$ and $^5D_0 \rightarrow ^7F_1$ bands.

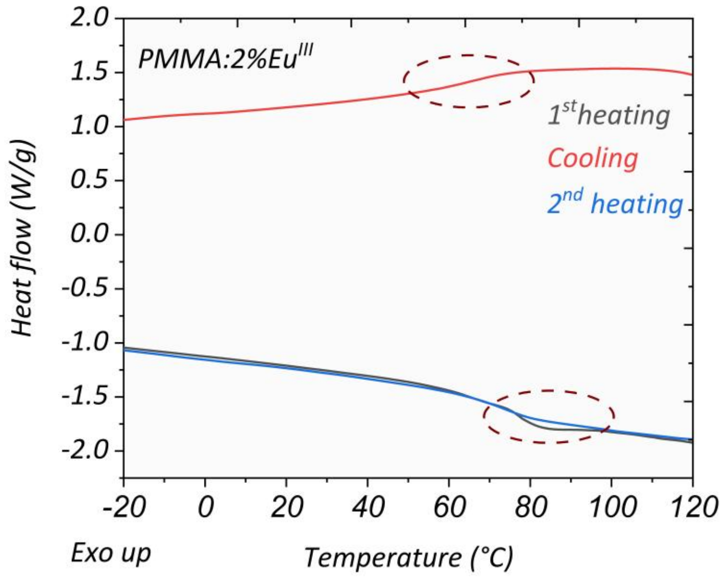

Figure S22. Differential scanning calorimetry (DSC) measurements undertaken for the PMMA:2% film under sequential heating and cooling cycles (thermal ramp of 10 °C min^{-1}). The red dashed circles represent the PMMA glass transition.

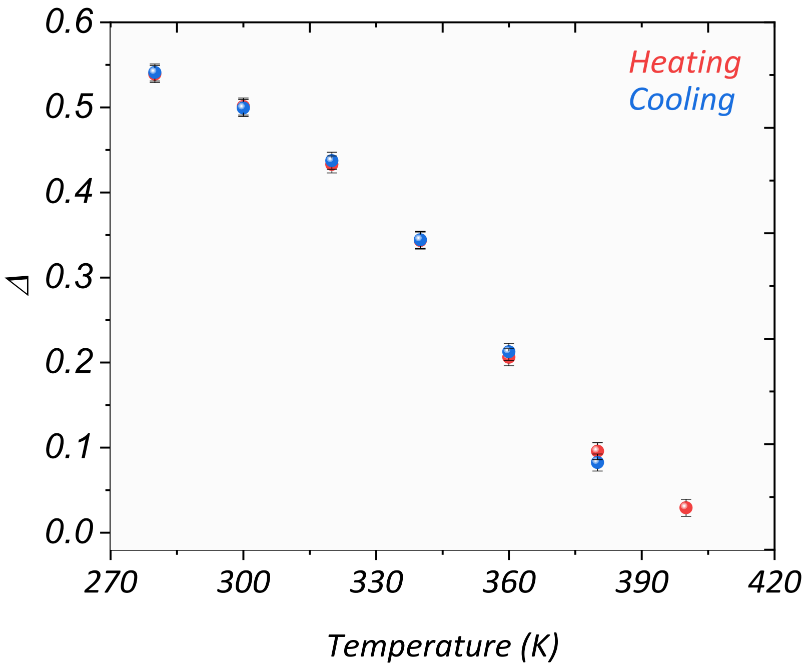

Figure S23. Dependency of the $^{5}D_0$ level lifetime on the temperature collected upon heating and cooling cycles; the $^{5}D_0$ level lifetime was used as the thermometric parameter (Δ) for the luminescence thermometry assay.

Table S13. State-of-the-art of luminescence temperature probes based on the Eu^{III} $^5D_0$ lifetime.

Material	Working temperature	S_r / % K^{-1}	Ref
**PMMA:2%Eu^{III}**	280 - 420	4.7	here
**$[L_1Eu]^-$**	298 - 323	1.8	[52]
**$[Eu(bzac)_3(H_2O)_2]$**	75 - 300	1.35	[53]
**$Eu(CPDK_{3-5})_3phen$**	298 - 348	1.2	[54]
**Eu-LnMOF, n = 1–3**	100 - 340	0.97	[55]
**$[Eu(hfa)_3(dpco)_2]$**	300 - 500	0.62	[56]

hfa: hexafluoroacetylacetonate; bzac: 1-Phenyl-1,3-butanedione; $CPDK_{3-5}$: 1-(4-(4-propylcyclohexyl) phenyl)octane-1,3-dione; phen: 1,10-phenathroline.

Supplementary references

[1] Estação Meteorológica Prof. Paulo Marques dos Santos (EM-14) – IAG/USP. Estação Meteorológica da Universidade de São Paulo. https://www.estacao.iag.usp.br/ (accessed 2026-06-23).

[2] Pinsky, M., & Avnir, D. (1998). Continuous symmetry measures. 5. The classical polyhedra. *Inorganic chemistry*, *37*(21), 5575-5582. https://doi.org/10.1021/ic9804925

[3] Hanuza, J., Ptak, M., Lisiecki, R., Janczak, J., Kwocz, A., Kucharska, E., ... & Macalik, L. (2018). Spectral and energetic transformation of femtosecond light impulses in the Eu3+ complex with dehydroacetic acid. *Journal of Luminescence*, *198*, 471-481. https://doi.org/10.1016/j.jlumin.2018.02.067

[4] Nie, D., Chen, Z., Bian, Z., Zhou, J., Liu, Z., Chen, F., ... & Huang, C. (2007). Energy transfer pathways in the carbazole functionalized β-diketonate europium complexes. *New Journal of Chemistry*, *31*(9), 1639-1646. https://doi.org/10.1039/B705666D

[5] Latva, M., Takalo, H., Mukkala, V. M., Matachescu, C., Rodríguez-Ubis, J. C., & Kankare, J. (1997). Correlation between the lowest triplet state energy level of the ligand and lanthanide (III) luminescence quantum yield. *Journal of Luminescence*, *75*(2), 149-169. https://www.sciencedirect.com/science/article/pii/S0022231397001130

[6] Moura Jr, R. T., Quintano, M., Santos-Jr, C. V., Albuquerque, V. A., Aguiar, E. C., Kraka, E., & Neto, A. N. C. (2022). Featuring a new computational protocol for the estimation of intensity and overall quantum yield in lanthanide chelates with applications to Eu (III) mercapto-triazole Schiff base ligands. *Optical Materials: X*, *16*, 100216. https://doi.org/10.1016/j.omx.2022.100216

[7] Moura Jr, R. T., Neto, A. N. C., Aguiar, E. C., Santos-Jr, C. V., de Lima, E. M., Faustino, W. M., ... & Malta, O. L. (2021). JOYSpectra: A web platform for luminescence of lanthanides. *Optical Materials: X*, *11*, 100080. https://doi.org/10.1016/j.omx.2021.100080

[8] Neto, A. N. C., Teotonio, E. E., de Sá, G. F., Brito, H. F., Legendziewicz, J., Carlos, L. D., ... & Malta, O. L. (2019). Modeling intramolecular energy transfer in lanthanide chelates: A critical review and recent advances. *Handbook on the physics and chemistry of rare earths*, *56*, 55-162. https://doi.org/10.1016/bs.hpcre.2019.08.001

[9] Moura Jr, R. T., Neto, A. N. C., Longo, R. L., & Malta, O. L. (2016). On the calculation and interpretation of covalency in the intensity parameters of 4f–4f transitions in Eu3+ complexes based on the chemical bond overlap polarizability. *Journal of Luminescence*, *170*, 420-430. https://doi.org/10.1016/j.jlumin.2015.08.016

[10] Neto, A. N. C., Moura Jr, R. T., Aguiar, E. C., Santos Jr, C. V., & de Medeiros, M. A. (2018). Theoretical study of geometric and spectroscopic properties of Eu (III) complexes with Ruhemann's Purple ligands. *Journal of Luminescence*, *201*, 451-459. https://doi.org/10.1016/j.jlumin.2018.05.014

[11] Neto, A. N. C., Moura Jr, R. T., & Malta, O. L. (2019). On the mechanisms of non-radiative energy transfer between lanthanide ions: centrosymmetric systems. *Journal of Luminescence*, *210*, 342-347. https://doi.org/10.1016/j.jlumin.2019.02.049

[12] Edvardsson, S., & Klintenberg, M. (1998). Role of the electrostatic model in calculating rare-earth crystal-field parameters. *Journal of alloys and compounds*, *275*, 230-233. https://doi.org/10.1016/S0925-8388(98)00309-0

[13] Smentek, L. (1998). Theoretical description of the spectroscopic properties of rare earth ions in crystals. *Physics Reports*, *297*(4), 155-237. https://doi.org/10.1016/S0370-1573(97)00077-X

[14] Freeman, A. J., & Desclaux, J. P. (1979). Dirac-Fock studies of some electronic properties of rare-earth ions. *Journal of Magnetism and Magnetic Materials*, *12*(1), 11-21. https://doi.org/10.1016/0304-8853(79)90328-7

[15] Malta, O. L., & Gouveia, E. A. (1983). Comment on the average energy denominator method in perturbation theory. *Physics Letters A*, *97*(8), 333-334. https://doi.org/10.1016/0375-9601(83)90655-2

[16] Malta, O. L., Ribeiro, S. J. L., Faucher, M., & Porcher, P. (1991). Theoretical intensities of 4f-4f transitions between stark levels of the Eu3+ ion in crystals. *Journal of Physics and Chemistry of Solids*, *52*(4), 587-593. https://doi.org/10.1016/0022-3697(91)90152-P

[17] Wybourne, B. G., & Smentek, L. (2007). *Optical spectroscopy of lanthanides: magnetic and hyperfine interactions*. CRC press. https://doi.org/10.1201/9781420006933

[18] Malta, O. L. (1982). A simple overlap model in lanthanide crystal-field theory. *Chemical Physics Letters*, *87*(1), 27-29. https://doi.org/10.1016/0009-2614(82)83546-X

[19] Malta, O. L. (1982). Theoretical crystal-field parameters for the YOCl: Eu3+ system. A simple overlap model. *Chemical Physics Letters*, *88*(3), 353-356. https://doi.org/10.1016/0009-2614(82)87103-0

[20] Neto, A. N. C., & Moura Jr, R. T. (2020). Overlap integrals and excitation energies calculations in trivalent lanthanides 4f orbitals in pairs Ln-L (L= Ln, N, O, F, P, S, Cl, Se, Br, and I). *Chemical Physics Letters*, *757*, 137884. https://doi.org/10.1016/j.cplett.2020.137884

[21] Judd, B. R. (1979). Ionic transitions hypersensitive to environment. *The Journal of Chemical Physics*, *70*(11), 4830-4833. https://doi.org/10.1063/1.437372

[22] Peacock, R. D. (2007). The intensities of lanthanide f↔ f transitions. In *Rare Earths* (pp. 83-122). Berlin, Heidelberg: Springer Berlin Heidelberg. https://doi.org/10.1016/B978-0-444-62735-3.00019-X

[23] Jørgensen, C. K., & Judd, B. R. (1964). Hypersensitive pseudoquadrupole transitions in lanthanides. *Molecular Physics*, *8*(3), 281-290. https://doi.org/10.1080/00268976400100321

---

[24] Mason, S. F., Peacock, R. D., & Stewart, B. (1975). Ligand-polarization contributions to the intensity of hypersensitive trivalent lanthanide transitions. *Molecular Physics*, *30*(6), 1829-1841. https://doi.org/10.1080/00268977500103321

[25] Malta, O. L., Batista, H. J., & Carlos, L. D. (2002). Overlap polarizability of a chemical bond: a scale of covalency and application to lanthanide compounds. *Chemical Physics*, *282*(1), 21-30. https://doi.org/10.1016/S0301-0104(02)00631-6

[26] Carlos, L. D., Malta, O. L., & Albuquerque, R. Q. (2005). A covalent fraction model for lanthanide compounds. *Chemical Physics Letters*, *415*(4-6), 238-242. https://doi.org/10.1016/j.cplett.2005.09.001

[27] Malta, O. L. (1997). Ligand—Rare-earth ion energy transfer in coordination compounds. A theoretical approach. *Journal of Luminescence*, *71*(3), 229-236. https://doi.org/10.1016/S0022-2313(96)00126-3

[28] Malta, O. L., & e Silva, F. G. (1998). A theoretical approach to intramolecular energy transfer and emission quantum yields in coordination compounds of rare earth ions. *Spectrochimica Acta Part A: Molecular and Biomolecular Spectroscopy*, *54*(11), 1593-1599. https://doi.org/10.1016/S1386-1425(98)00086-9

[29] Longo, R., e Silva, F. R. G., & Malta, O. L. (2000). A theoretical study of the energy-transfer process in [Eu⊂ bpy. bpy. bpy] 3+ cryptates: a ligand-to-metal charge-transfer state?. *Chemical Physics Letters*, *328*(1-2), 67-74. https://doi.org/10.1016/S0009-2614(00)00898-8

[30] Malta, O. L. (2008). Mechanisms of non-radiative energy transfer involving lanthanide ions revisited. *Journal of non-crystalline solids*, *354*(42-44), 4770-4776. https://doi.org/10.1016/j.jnoncrysol.2008.04.023

[31] Carnall, W. T., Crosswhite, H., & Crosswhite, H. M. (1977). *Energy level structure and transition probabilities in the spectra of the trivalent lanthanides in* LaF_3 (No. ANL--78-XX-95). Argonne National Lab.(ANL), Argonne, IL (United States). https://doi.org/10.2172/6417825

[32] e Silva, F. R., & Malta, O. L. (1997). Calculation of the ligand–lanthanide ion energy transfer rate in coordination compounds: contributions of exchange interactions. *Journal of alloys and compounds*, *250*(1-2), 427-430. https://doi.org/10.1016/S0925-8388(96)02563-7

[33] Kasprzycka, E., Neto, A. N. C., Trush, V. A., Jerzykiewicz, L., Amirkhanov, V. M., Malta, O. L., ... & Gawryszewska, P. (2020). How minor structural changes generate major consequences in photophysical properties of RE coordination compounds; resonance effect, LMCT state. *Journal of Rare Earths*, *38*(5), 552-563. https://doi.org/10.1016/j.jre.2020.02.001

[34] Moura Jr, R. T., Neto, A. N. C., Aguiar, E. C., Santos-Jr, C. V., de Lima, E. M., Faustino, W. M., ... & Malta, O. L. (2021). JOYSpectra: A web platform for luminescence of lanthanides. *Optical Materials: X*, *11*, 100080. https://doi.org/10.1016/j.omx.2021.100080

[35] Malta, O. L. (1997). Ligand—Rare-earth ion energy transfer in coordination compounds. A theoretical approach. *Journal of Luminescence*, *71*(3), 229-236. https://doi.org/10.1016/S0022-2313(96)00126-3

[36] Malta, O. L., & e Silva, F. G. (1998). A theoretical approach to intramolecular energy transfer and emission quantum yields in coordination compounds of rare earth ions. *Spectrochimica Acta Part A: Molecular and Biomolecular Spectroscopy*, *54*(11), 1593-1599. https://doi.org/10.1016/S1386-1425(98)00086-9

[37] Longo, R., e Silva, F. R. G., & Malta, O. L. (2000). A theoretical study of the energy-transfer process in [Eu⊂ bpy. bpy. bpy] 3+ cryptates: a ligand-to-metal charge-transfer state?. *Chemical Physics Letters*, *328*(1-2), 67-74. https://doi.org/10.1016/S0009-2614(00)00898-8.
[38] Malta, O. L. (2008). Mechanisms of non-radiative energy transfer involving lanthanide ions revisited. *Journal of non-crystalline solids*, *354*(42-44), 4770-4776. https://doi.org/10.1016/j.jnoncrysol.2008.04.023.
[39] Graffion, J., Cojocariu, A. M., Cattoën, X., Ferreira, R. A., Fernandes, V. R., André, P. S., ... & Bartlett, J. R. (2012). Luminescent coatings from bipyridine-based bridged silsesquioxanes containing Eu 3+ and Tb 3+ salts. *Journal of Materials Chemistry*, *22*(26), 13279-13285. https://doi.org/10.1039/C2JM31289A
[40] Griffini, G., Levi, M., & Turri, S. (2013). Novel crosslinked host matrices based on fluorinated polymers for long-term durability in thin-film luminescent solar concentrators. *Solar energy materials and solar cells*, *118*, 36-42. https://doi.org/10.1016/j.solmat.2013.05.041
[41] Meinardi, F., McDaniel, H., Carulli, F., Colombo, A., Velizhanin, K. A., Makarov, N. S., ... & Brovelli, S. (2015). Highly efficient large-area colourless luminescent solar concentrators using heavy-metal-free colloidal quantum dots. *Nature nanotechnology*, *10*(10), 878-885. https://doi.org/10.1016/j.solmat.2013.05.041
[42] Currie, M. J., Mapel, J. K., Heidel, T. D., Goffri, S., & Baldo, M. A. (2008). High-efficiency organic solar concentrators for photovoltaics. *Science*, *321*(5886), 226-228. https://www.science.org/doi/full/10.1126/science.1158342
[43] Wang, Y., Xie, G., Chen, J., Zhang, X., Chen, C., Yin, J., & Li, H. (2022). Visible-light excitable, highly transparent and luminescent films with an ultrahigh loading of a europium (III) complex. *Journal of Materials Chemistry C*, *10*(33), 11924-11930. https://doi.org/10.1039/D2TC02266D
[44] Zhou, Y., Benetti, D., Fan, Z., Zhao, H., Ma, D., Govorov, A. O., ... & Rosei, F. (2016). Near infrared, highly efficient luminescent solar concentrators. *Advanced Energy Materials*, *6*(11), 1501913. https://doi.org/10.1002/aenm.201501913
[45] Meinardi, F., Colombo, A., Velizhanin, K. A., Simonutti, R., Lorenzon, M., Beverina, L., ... & Brovelli, S. (2014). Large-area luminescent solar concentrators based on 'Stokes-shift-engineered'nanocrystals in a mass-polymerized PMMA matrix. *Nature photonics*, *8*(5), 392-399. https://www.nature.com/articles/nphoton.2014.54
[46] Mateen, F., Lee, S. Y., & Hong, S. K. (2020). Luminescent solar concentrators based on thermally activated delayed fluorescence dyes. *Journal of Materials Chemistry A*, *8*(7), 3708-3716. https://doi.org/10.1039/C9TA13312G
[47] Freitas, V. T., Fu, L., Cojocariu, A. M., Cattoen, X., Bartlett, J. R., Le Parc, R., ... & Carlos, L. D. (2015). Eu3+-based bridged silsesquioxanes for transparent luminescent solar concentrators. *ACS applied materials & interfaces*, *7*(16), 8770-8778. https://doi.org/10.1021/acsami.5b01281
[48] Banal, J. L., White, J. M., Ghiggino, K. P., & Wong, W. W. (2014). Concentrating aggregation-induced fluorescence in planar waveguides: a proof-of-principle. *Scientific reports*, *4*(1), 4635. https://www.nature.com/articles/srep04635
[49] Kaniyoor, A., McKenna, B., Comby, S., & Evans, R. C. (2016). Design and response of high-efficiency, planar, doped luminescent solar concentrators using organic–inorganic di-ureasil

waveguides. *Advanced Optical Materials*, *4*(3), 444-456. https://doi.org/10.1002/adom.201500412

[50] Mateen, F., Ali, M., Lee, S. Y., Jeong, S. H., Ko, M. J., & Hong, S. K. (2019). Tandem structured luminescent solar concentrator based on inorganic carbon quantum dots and organic dyes. *Solar Energy*, *190*, 488-494. 10.1016/j.solener.2019.08.045

[51] Brites, C. D. S., Millán, A., Carlos, L. D. (2016). Lanthanides in luminescent thermometry. In *Handbook on the physics and chemistry of rare earths* (Vol. 49, pp. 339-427). Elsevier. https://doi.org/10.1016/bs.hpcre.2016.03.005

[52] Cheung, T. L., Ju, Z., Zhang, W., Parker, D., & Deng, R. (2024). Mechanistic Investigation of Sensitized Europium Luminescence: Excited State Dynamics and Luminescence Lifetime Thermometry. *ACS Applied Materials & Interfaces*, *16*(33), 43933-43941. https://doi.org/10.1021/acsami.4c06899

[53] Gálico, D. A., Mazali, I. O., & Sigoli, F. A. (2017). Nanothermometer based on intensity variation and emission lifetime of europium (III) benzoylacetonate complex. *Journal of Luminescence*, *192*, 224-230. https://doi.org/10.1016/j.jlumin.2017.06.062

[54] Lapaev, D. V., Nikiforov, V. G., Lobkov, V. S., Knyazev, A. A., & Galyametdinov, Y. G. (2018). Reusable temperature-sensitive luminescent material based on vitrified film of europium (III) β-diketonate complex. *Optical Materials*, *75*, 787-795. https://doi.org/10.1016/j.optmat.2017.11.042

[55] Wang, C., Xi, P., Shi, W., Yan, X., Zhang, C., & Cheng, B. (2025). Structure, fluorescence enhancement mechanism and luminescence thermometry functions of multi-podal benzoate Eu (III) luminescent materials. *Materials Research Bulletin*, 113627. https://doi.org/10.1016/j.materresbull.2025.113627

[56] Kitagawa, Y., Kumagai, M., Nakanishi, T., Fushimi, K., & Hasegawa, Y. (2020). The role of π–f orbital interactions in Eu (III) complexes for an effective molecular luminescent thermometer. *Inorganic Chemistry*, *59*(9), 5865-5871. https://doi.org/10.1021/acs.inorgchem.9b03492
